# Smart Gas Sensors: Recent Developments and Future Prospective

**DOI:** 10.1007/s40820-024-01543-w

**Published:** 2024-11-04

**Authors:** Boyang Zong, Shufang Wu, Yuehong Yang, Qiuju Li, Tian Tao, Shun Mao

**Affiliations:** 1 College of Environmental Science and Engineering, Biomedical Multidisciplinary Innovation Research Institute, Shanghai East Hospital, State Key Laboratory of Pollution Control and Resource Reuse, Tongji University, 1239 Siping Road, Shanghai, 200092 People’s Republic of China; 2 Shanghai Institute of Pollution Control and Ecological Security, Shanghai, 200092 People’s Republic of China; 3 Microbiome Medicine Center, Department of Laboratory Medicine, Zhujiang Hospital, Southern Medical University, Guangzhou, 510280 People’s Republic of China

**Keywords:** Smart gas sensor, Electronic sensor, Optoelectronic sensor, Flexible and wearable sensor, Artificial intelligence

## Abstract

Gas sensor is an indispensable part of modern society with wide applications in environmental monitoring, healthcare, food industry, public safety, etc. With the development of sensor technology, wireless communication, smart monitoring terminal, cloud storage/computing technology, and artificial intelligence, smart gas sensors represent the future of gas sensing due to their merits of real-time multifunctional monitoring, early warning function, and intelligent and automated feature. Various electronic and optoelectronic gas sensors have been developed for high-performance smart gas analysis. With the development of smart terminals and the maturity of integrated technology, flexible and wearable gas sensors play an increasing role in gas analysis. This review highlights recent advances of smart gas sensors in diverse applications. The structural components and fundamental principles of electronic and optoelectronic gas sensors are described, and flexible and wearable gas sensor devices are highlighted. Moreover, sensor array with artificial intelligence algorithms and smart gas sensors in “Internet of Things” paradigm are introduced. Finally, the challenges and perspectives of smart gas sensors are discussed regarding the future need of gas sensors for smart city and healthy living.

**Supplementary Information:**

The online version contains supplementary material available at 10.1007/s40820-024-01543-w.

## Highlights

- Recent developments of advanced electronic and optoelectronic gas sensors are introduced.
- Sensor array with artificial intelligence algorithms and smart gas sensors in “Internet of Things” paradigm are highlighted.
- Applications of smart gas sensors in environmental monitoring, medical and healthcare applications, food quality control, and public safety are described.

**Supplementary Information:**

The online version contains supplementary material available at 10.1007/s40820-024-01543-w.

## Graphical abstract

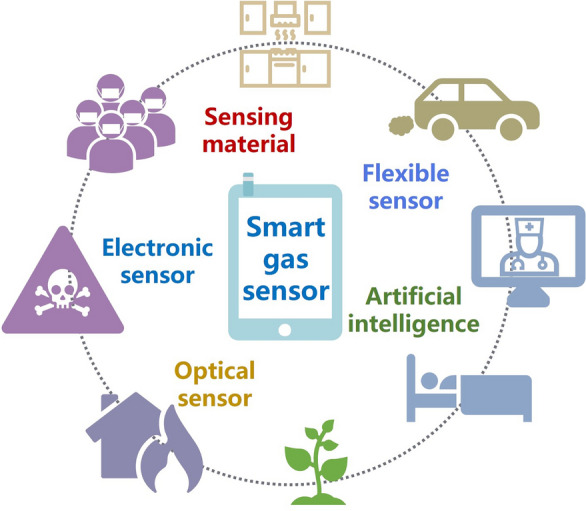

## Introduction

**Fig. 1:**
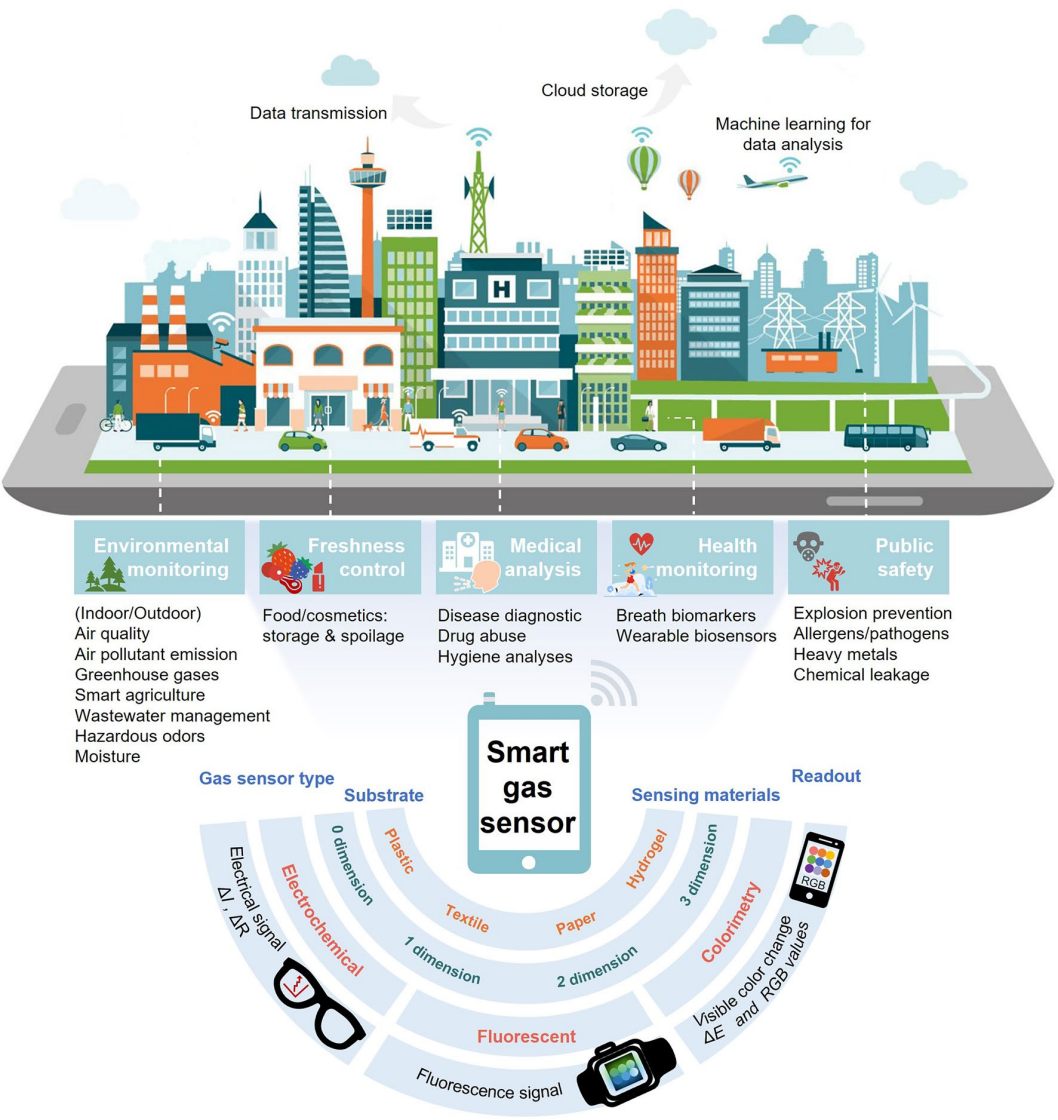
Smart gas sensors with widespread use in multiple scenes of human life. Adapted illustration 81383831. Copyright Elenabsl Dreamstime.com

Gas sensor is a transducer that converts the interaction between gaseous analyte and sensing material into a suitable form amenable for further processing, providing gas composition and concentration information [1, 2]. In the past decades, gas sensors have become an indispensable part of modern life (Fig. 1) with a broad range of applications in atmospheric and indoor monitoring, medical and healthcare, food industry, public safety, chemical production, etc. Together with the boom of the Internet of the Things (IoT) technology and rising demand for smart applications, smart gas sensors emerge as required. Smart gas sensor, also called intelligent gas sensor or digital gas sensor, is a module integration that is with sensor-centered, integrated with communication technology and artificial intelligence technology, and in the form of portable sensor [3], flexible and wearable sensor [4–6], and sensor array [7, 8]. Smart gas sensors are expected to work in future digital home [9], early stage diagnosis [10–12], noninvasive medical treatment [13], fitness tracking [14], food-quality assessment [15], remote warning of gas leakage [16], individual authentication [17], epidemic early warning [18], touchless interactive panel [19], visible industrial safety alert [20], living-plant healthcare [21], etc.

**Fig. 2:**
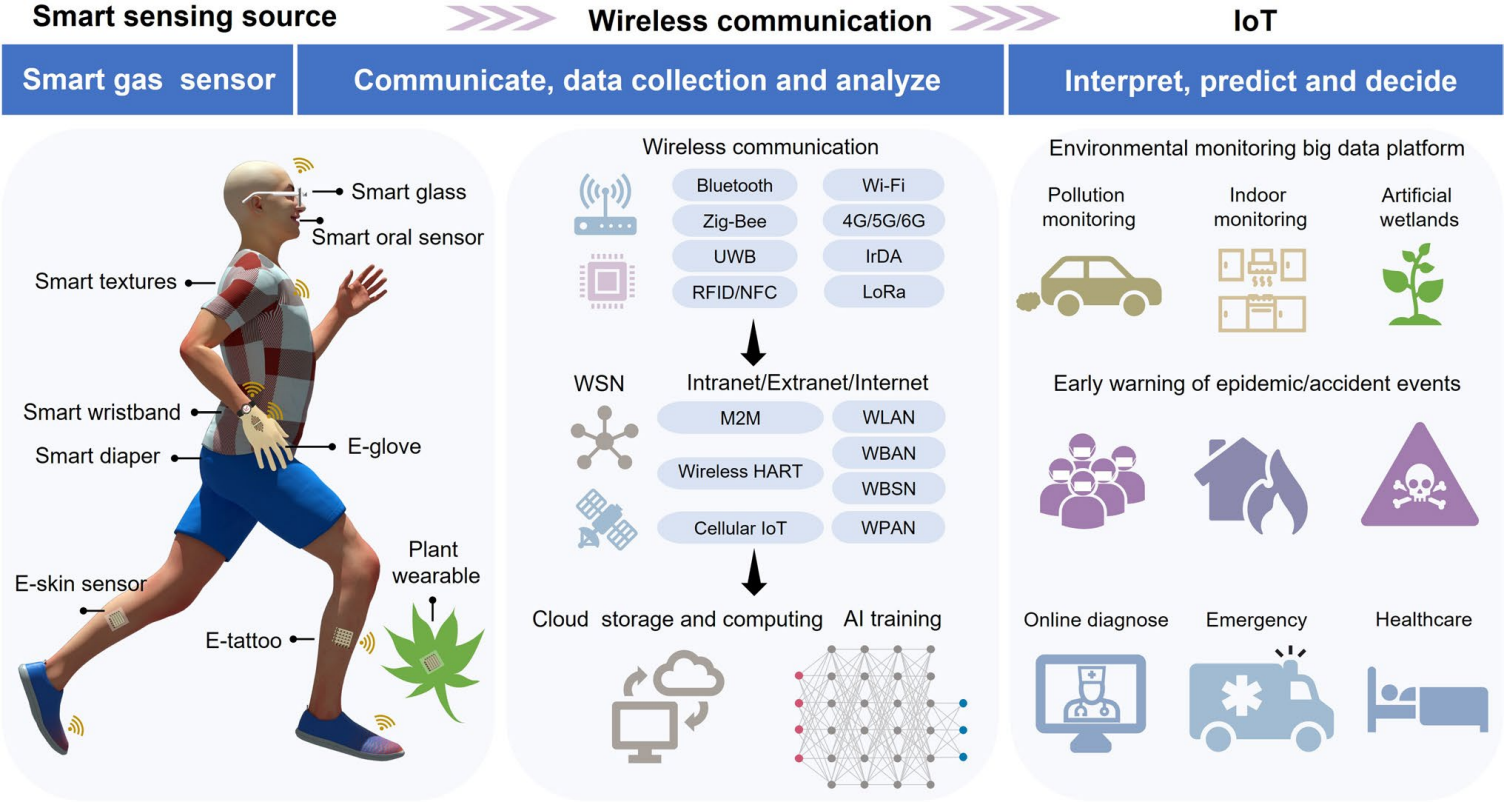
Full-spectrum operation procedures of smart gas sensors

In the past decade, smart gas sensors, especially portable and wearable gas sensors, have become a high-efficient and flexible tool in precise analysis field that is restricted by personnel perception infeasibility, complex pretreatment procedures, and the inevitable use of harmful chemical reagents [22, 23]. The electronics industry has promoted the integration of miniaturized sensing chips with standard electronic components, giving rise to wearable 1.0, mainly in the rigid form of smart phone, smart glasses, smart wristband, smart watch, etc. In recent years, powered by the growing market demand for biometric information and wearable bio-diagnostics, along with the advancement of IoT technology, big data, artificial intelligence (AI), robotics, current rigid wearable 1.0 have moved to the next-generation wearable 2.0 era. Future wearables will surpass the limits of current rigid wafers and planar circuit platform technologies and be soft, skin-attachable, stretchable, bendable, twistable, rollable, in the forms of textiles, patches, tattoos, even tissue hybrids [4, 5]. The IoT ecology chain consists of (1) flexible and wearable sensors for sensing and signal transduction; (2) wireless communication for transforming signal and sending data to cloud storage and computing; and (3) AI training andd warning system for analyzing, interpreting, predicting, and generating early alert (Fig. 2). Non-wearable smart gas sensors and sensor arrays have been employed in high-precise exhaust emission monitoring, hazardous and toxic gases leakage detection for early alarms, mobile environmental monitoring for enforcement; while smart wearables have emerged in non-invasive diagnosis and smart agriculture, and tend to evolve in online healthcare and early warning of epidemic events through IoT.

**Fig. 3:**
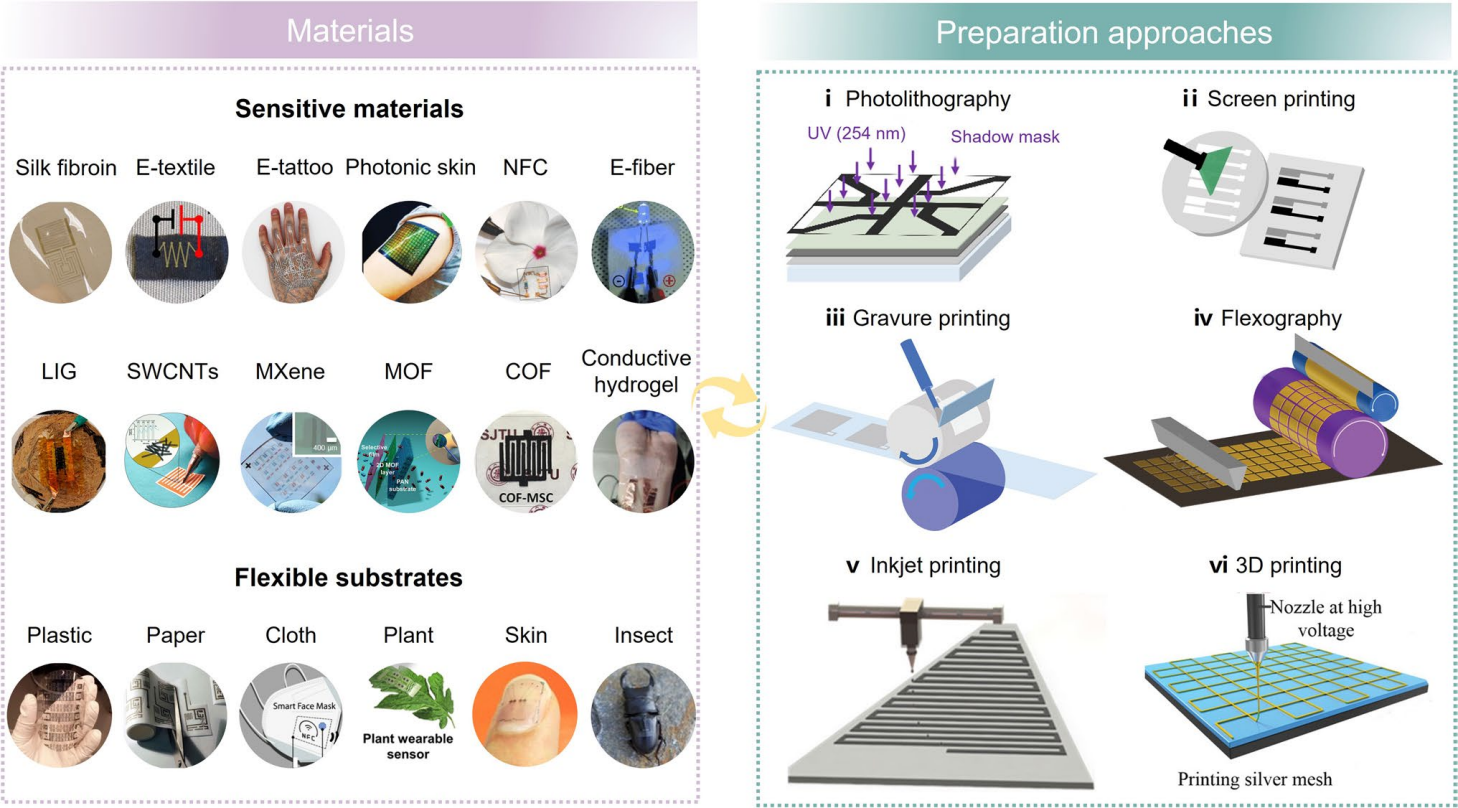
Substrate and conductive materials and the fabrication methods for flexible and wearable sensing devices. Image for “Silk fibroin,” reproduced with permission from Ref. [69]. Copyright 2012, Springer Nature. Image for “Electronic textile” (E-textile), reproduced with permission from Ref. [70]. Copyright 2017, American Chemical Society. Image for “Electronic tattoo” (E-tattoo), reproduced with permission from Ref. [71]. Copyright 2021, American Association for the Advancement of Science. Image for “Photonic skin,” reproduced with permission from Ref. [47]. Copyright 2019, Wiley–VCH. Image for “Nanofibril cellulose” (NFC) thin film, reproduced with permission from Ref. [72]. Copyright 2014, Wiley–VCH. Image for “Electronic fiber” (E-fiber), reproduced with permission from Ref. [73]. Copyright 2013, Wiley–VCH. Image for “Laser-induced graphene” (LIG), reproduced with permission from Ref. [52]. Copyright 2018, American Chemical Society. Image for “Drawned single-walled carbon nanotubes” (Drawned SWCNTs), reproduced with permission from Ref. [54]. Copyright 2012, Wiley–VCH. Image for “MXene electrode array,” reproduced with permission from Ref. [74]. Copyright 2019, American Chemical Society. Image for “Metal–Organic Framework” (2D MOF) thin film, reproduced with permission from Ref. [75]. Copyright 2018, American Chemical Society. Image for “Covalent–Organic Framework” (2D COF) thin film, reproduced with permission from Ref. [76]. Copyright 2019, Wiley–VCH. Image for “Conductive hydrogel,” Reproduced with permission from Ref. [77]. Copyright 2023, Wiley–VCH. Image for plastic substrate, reproduced with permission from Ref. [78]. Copyright 2015, American Chemical Society. Image for “Paper-based electronic circuits,” reproduced with permission from Ref. [79]. Copyright 2009, Wiley–VCH. Images for cloth substrate, reproduced with permission from Ref. [80]. Copyright 2022, Springer Nature. Images for plant leaf substrate, reproduced with permission from Ref. [21]. Copyright 2023, Springer Nature. Images for life form (*e.g.*, fingernail, insect) substrate, reproduced with permission from Ref. [81]. Copyright 2014, American Chemical Society. Image for “Photolithography fabrication technology,” reproduced with permission from Ref. [27]. Copyright 2017, Wiley–VCH. Image for “Screen printing fabrication technology,” reproduced with permission from Ref. [32]. Copyright 2021, Shanghai Jiao Tong Univ Press. Image for “Gravure printing fabrication technology,” reproduced with permission from Ref. [36]. Copyright 2020, Wiley–VCH. Image for “Inkjet printing technology,” reproduced with permission from Ref. [35]. Copyright 2020, American Chemical Society. Image for “3D printing technology,” reproduced with permission from Ref. [82]. Copyright 2021, Wiley–VCH. Image for “Flexography fabrication technology,” reproduced with permission from Ref. [83]. Copyright 2023, Wiley–VCH

The soft electronic circuits are the core components in portable gas sensor. There are three strategies to achieve stretchable and wearable electrodes: (1) assembly of the rigid inorganic semiconducting material/soft organic semiconducting material and circuits (Au/Ag/Cu and conductive ink) onto the flexible substrate; (2) directly bond thin conductive material with low Young’s modulus onto the flexible substrate; and (3) prepare the conductors that are inherently stretchable, for instance, mix the conductive material with the flexible substrate. Common flexible electrode fabrication technologies include photolithography [24–27] (e.g., physical vapor deposition [28], chemical vapor deposition [29], magnetron sputtering [30], electron-beam evaporation [31]), screen printing [32], gravure printing [33], inkjet printing [34, 35], and 3D printing [36] (Fig. 3). Diverse flexible substrates, including plastic polymers [37], cellulose paper [38, 39], silk [40–43], even skin [44–46], with different levels of roughness and surface energies [47], influence the mechanical stretchability and adaptability of flexible and wearable electronic devices. Besides, inorganic semiconducting materials including metal oxides (*e.g.*, ZnO [48], SnO_2_ [49], WO_3_ [50], Sn-doped- Bi_2_O_2_CO_3_ [51]), graphene [52, 53], carbon nanotubes (CNTs) [54], transition metal dichalcogenides (*e.g.*, MoS_2_ [55] and WS_2_ [56]), MXene (*e.g.*, Ti_3_C_2_T_*x*_ [57] and V_4_C_3_T_*x*_ [58]), phosphorene (*e.g.*, black phosphorus [59] and violet phosphorus [60]), organic semiconducting materials including conductive metal–organic framework (*e.g.*, Cu_3_(HITP)_2_ [61] and Ni_3_(HHTP)_2_ [62]), covalent organic framework (*e.g.*, pyrene COF [63]), hydrogen-bonded organic framework (*e.g.*, HOF-FJU-1 [64] and 8PN [65]), hydrogel [66–68] as well as other conductive polymers can either be used as electrode or sensitive material.

Gas sensors can be categorized into electrically transduced sensor and optically transduced sensor [84, 85]. Electrically transduced sensors (electronic sensors) have gained a key role in the gas sensing field, due to integrability with wireless communication and microprocessor modules, compatibility with standard electronic components, operability, portability, real-time monitoring, and quick analysis [85–89]. The electrical property (*e.g.*, capacitance, impedance, resistance, current, and voltage) variation of a conductive sensing material can be transformed into a readable electronic signal and reflect the information (species and concentration) of the gas. The electronic gas sensors may have covalent or noncovalent interface interaction between the sensing material and the gas molecule [90–92]. Representative electronic gas sensors include field-effect transistor (FET), capacitor, chemiresistor, and electrochemical sensor [93–96]. The conductive sensing materials in electronic sensor mainly contain semiconductors and conducting polymers in multi-dimensional forms. The technological advancement in flexible design and feasible functionalization of sensing materials leads to flexible electronic gas sensors, which achieve highly sensitive (ppb-level detection) detection of target analyte. Currently, the discrimination of the gas analogs with similar chemical structures and physical properties and the recognition of specific target analyte in the mixture gas environment remain as a thorny challenge for the application of electronic gas sensors.

Optoelectronic sensors provide a visual identifiability platform for gas detection with highly selective and discriminatory responses by fluorimetry or colorimetry methods [97–101]. Unlike electronic sensor with a physical electronic property change as the sensing signal, optoelectronic sensors mainly employ chemical characteristics variation induced by the target gas and the sensing material. The visual fluorescence or color response upon exposure to target gas reflects the optical property variation in the sensing material during the detection process [98, 102–106]. However, the inappreciable level of sensitivity caused by gas analyte with relatively low sensitivity is still a challenge to optoelectronic gas sensors [97]. Besides, accurate recognition and quantitative analysis of unknown gas in real-world application are a common challenge for both electronic and optoelectronic gas sensors.

In this review article, the working principle, structure design, signal transduction, detection performance, and recent breakthroughs of smart electronic and optoelectronic gas sensors in diverse practical application scenarios are summarized. The strategies for enhancing selectivity, accuracy, and sensitivity by constructing sensor array, machine-learning (ML) algorithm training, and ingenious engineering of the applied sensing material are introduced. This review provides new conception of remote and in-field gas sensing by wirelessly transformation network technology and AI-enabled data analysis, which serves as the power source for the IoT. The challenges facing the employment of smart gas sensors and the future development trends are also discussed.

## Logical Structure and Working Mechanism of Gas Sensors

The current section focuses on the gas sensors based on electrical and optical principles that sustain considerable scientific interests. The specialty area includes, but is not limited to, field-effect transistors (FETs), chemiresistors, capacitors, diodes, electrochemical sensors, colorimetric and fluorescent detectors [85, 107]. Other sensing methods such as non-dispersive infrared analyzers, photo-ionization detectors, fiber waveguide sensors, and interferometric sensors are not discussed in this review.

### Electronic Gas Sensors

**Fig. 4:**
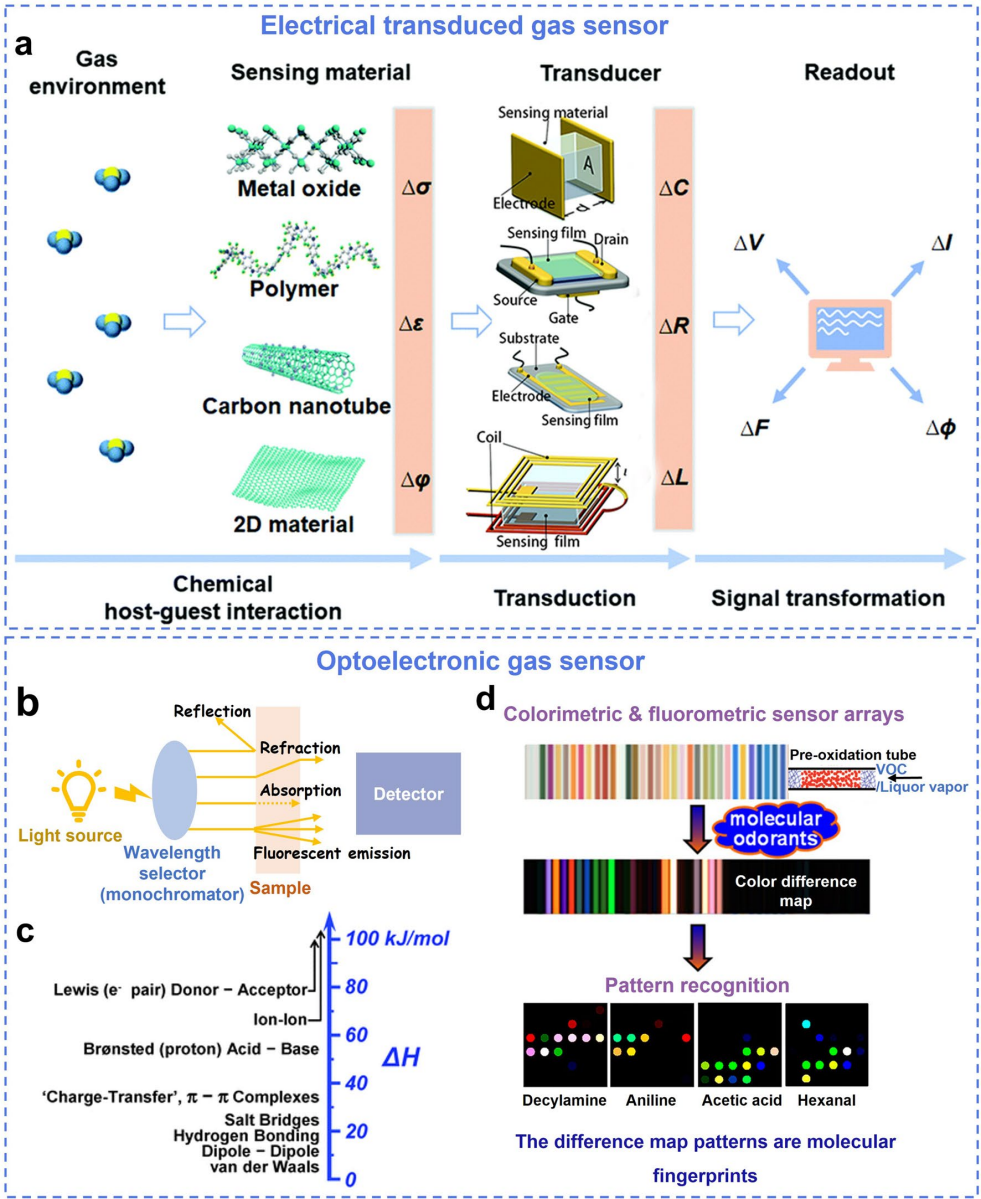
Scheme mechanism of electrochemical and optoelectronic gas sensor. **a** Schematic illustration of the electrical gas sensors (Type of the transducer from top to bottom: capacitor, FET, chemiresistor, inductive gas sensor). Reproduced with permission from Ref. [39]. Copyright 2020, American Chemical Society. **b** Scheme of a general spectroscopic setup. Reproduced with permission from Ref. [84]. Copyright 2018, American Chemical Society. **c** The range of physical or chemical intermolecular interactions to probe VOC signals, from the weakest van der Waals to the very strong covalent and ionic bonds. Reproduced with permission from Ref. [98]. Copyright 2013, American Chemical Society. **d** Schematic illustration of the sensor array. Reproduced with permission from Ref. [84]. Copyright 2018, American Chemical Society. The pre-oxidation tube. Reproduced with permission from Ref. [118]. Copyright 2017, American Chemical Society. The difference map patterns of decylamine, aniline, acetic acid and hexanal are examples of molecular fingerprints. Reproduced with permission from Ref. [119]. Copyright 2020, American Chemical Society

Electronic gas sensors comprise two main components: the sensing material and the transducer (Fig. 4a) [85]. The sensing material that is exposed to ambient environment will interact with target analyte mostly through physical adsorption. The gas–solid interaction induces a change in its physical properties (*e.g.*, variance of conductivity (Δ*σ*), permittivity (Δ*ε*), work function (Δ*φ*)). The electronic components in the electronic gas sensors (including FETs, resistors, capacitors, inductors) convert the corresponding physical quantities into the electrical measurable parameters (*e.g.*, capacitance (Δ*C*), resistance (Δ*R*), inductance (Δ*L*)), and the final sensing signal is typically in the form of current (Δ*I*) and voltage (Δ*U*) variations [85, 89, 92, 93, 108]. The characteristics of different types of transducers in electronic gas sensors are listed in Table 1. The key sensing parameters and definitions of gas sensors are listed in Table S1.

**Table 1:** Characteristics of different types of transducers in electronic gas sensors [39]

Transduction type	Advantages	Disadvantages
Chemiresistor	Simple configuration and working principle	Susceptible to environmental perturbations, restricted by single type of output (*e.g.*, resistance or current), high operating temperature, cross-sensitivity, aging and drifting
Field-effect transistor	Diverse types of output signals (*e.g.*, drain-source current, threshold voltage, sub-threshold swing)	Susceptible to environmental perturbations, cross-sensitive to gas with high structural and property similarities, underperforming recovery and long-term stability
Capacitor	Capability of additional measurement than chemiresistor, allowing for better selectivity and reliability	Susceptible to environmental cleanliness, edge effect, and parasitic capacitance
Inductor	Can be magnetically coupled with an external coil for wireless detection	Less common due to relatively complex circuit configuration

In recent decades, the miniaturization and integration of conventional silicon-based rigid sensors gave rise to smart devices (*e.g.*, smart phone and smart watch) for impressive user experience. Thereafter, emerging flexible and wearable electronic sensors further enable advances in skin patches, electronic tattoos, and smart clothing, where traditional rigid electronic sensors restrict their usage. Flexible electronic sensors are usually manufactured using low-cost materials and large-scale processes like printing (Fig. 3). In this context, electronic gas sensors have been promoted by synergistic breakthroughs in sensing material and the flexible substrate. Substrates are not limited to plastic foil [78], paper [79], textile [81], hydrogel [109], which can either be bendable, rollable, foldable, stretchable, twistable and conformable [4]. Chen et al. defined flexible sensors as those can withstand mechanical deformation (> 10 m^−1^ bending curvature or > 1% strain) without device failure or significant alteration in sensing performances [4]. Hence, the effective electronic gas sensors in both rigid and flexible form own at least twofold strength [85, 92]. First, the sensing materials should provide a large exposed surface and selective binding sites for the covalent or noncovalent material–analyte interaction and respond to the interaction by changing their electrical properties, such as conductivity, work function, and electrical permittivity. Second, the transducer (usually refers to standard electronic components including chemiresistor, FET, capacitor, inductor, etc*.*) entails a conversion of sensing event into a measurable and readable electrical signal (change in resistance, current, magnitude, or frequency). Besides, the flexible and wearable substrate requires good mechanical flexibility to adapt multiple particle application scenes.

### Optoelectronic Gas Sensors

Optoelectronic gas sensors are generally based on various optical principles such as absorption, scattering, diffraction, reflectance, refraction, luminescence (*e.g.*, photo-, chemi-, electrochemi-, and bio-luminescence) (Fig. 4b) [98]. Of these, colorimetric and fluorescent sensors are widely reported in gas sensing based on the intermolecular interactions between the chromophore or fluorophore with the target analytes [84]. Optoelectronic gas sensors based on chemoresponsive colorants probe the chemical sensing signals of analytes, rather than physical properties, providing impressive discrimination among very similar analytes, which therefore effectively overcome the limitation of traditional physisorption or nonspecific chemical interactions. Optoelectronic selectivity and distinguishing capability are the consequence of intermolecular interactions from the very weak van der Waals to the strong covalent or ionic bonds (Fig. 4c) [98].

Optoelectronic gas sensors comprise four key elements: a light source (*e.g.*, visible or ultraviolet light), a wavelength selection device, a substrate, and a detector sensitive to the interesting wavelength (Fig. 4b) [98]. Combining array-based techniques that use a chemically diverse set of cross-reactive sensors with novel digital imaging methods, such optoelectronic gas sensor array (which also called optoelectronic noses or tongues) can produce an exquisite visual fingerprinting for target odorants through color difference map patterns, and further enhanced olfactory specificity from pattern recognition of the responses (Fig. 4d). Generally, optoelectronic gas sensing materials incorporated in diverse substrates, including paper substrate [110], films [111], hydrogels [112], silica gel [113], and matrices [114], similar to flexible electronics, enrich their practical applications.

Color models (or color spaces) provide a mathematical structure for representing color change, converting complex color change information into measurable and processable form [115]. Common color models include CIELAB (Commission Internationale de I’Eclariage), RGB (Red, Green, Blue), HSV (Hue, Saturation, Value), CMYK (Cyan, Magenta, Yellow, Black), YIQ (Luminance, In-Phase, Quadrature-Phase), YUV (Luminance, Chrominance, Chroma) and YC_b_C_r_ (Luminance, color-difference of blue, color-difference of red), among which CIELAB and RGB have been used extensively in colorimetric gas sensor. The color models are used for chart the relationship between color changes and analyte concentration owing to their own way to extract color information.

The RGB color model is based on the intensity changes of red, green and blue colors (each of whose range 0–255). RGB color model is widely used in color sensing, with certain color channels aligning well with sample absorbance peak to improve measurement accuracy [116]. Various methodological methods including the calculation of Euclidean distance between colors and the calculation of ratios (*e.g.*, B/R, R/B, G/B, R/G) or more complex combinations (e.g., $\frac{\sqrt{{\text{R}}^{2}\text{+}{\text{G}}^{2}\text{+}{\text{B}}^{2}}}{3}$, (B-G)/R, etc*.*) are used for color analysis; and few studies merely use single channel to increase resolution [115]. The CIELAB color model is based on the change of lightness (L) and A/B (red/green) color channels. CIELAB model is more intuitive for color perception in human vision and not so susceptible to varying devices and environment (such as lightening conditions), thus, it is widely used in colorimetry sensing. CIELAB model uses Cartesian coordinates for a 3D spatial representation to improve uniformity in color perception and employs Euclidean distance (Δ*E*) within the color space to quantify the sensing response [117]. The HSV color model is based on the change of hue, saturation, and brightness value. HSV’s maximum value reflects the color’s brightness under direct light, and HSV’s consistence across various lighting conditions makes it preferred than the RGB model.

## Smart Gas Sensor Applications

The rapid development of efficient, simple and integrated smart electronic and optoelectronic gas sensors has broaden their applications in multiple fields such as environmental air pollutants monitoring, medical diagnosis, food spoilage detection, and public safety warning.

### Environmental Monitoring

**Fig. 5:**
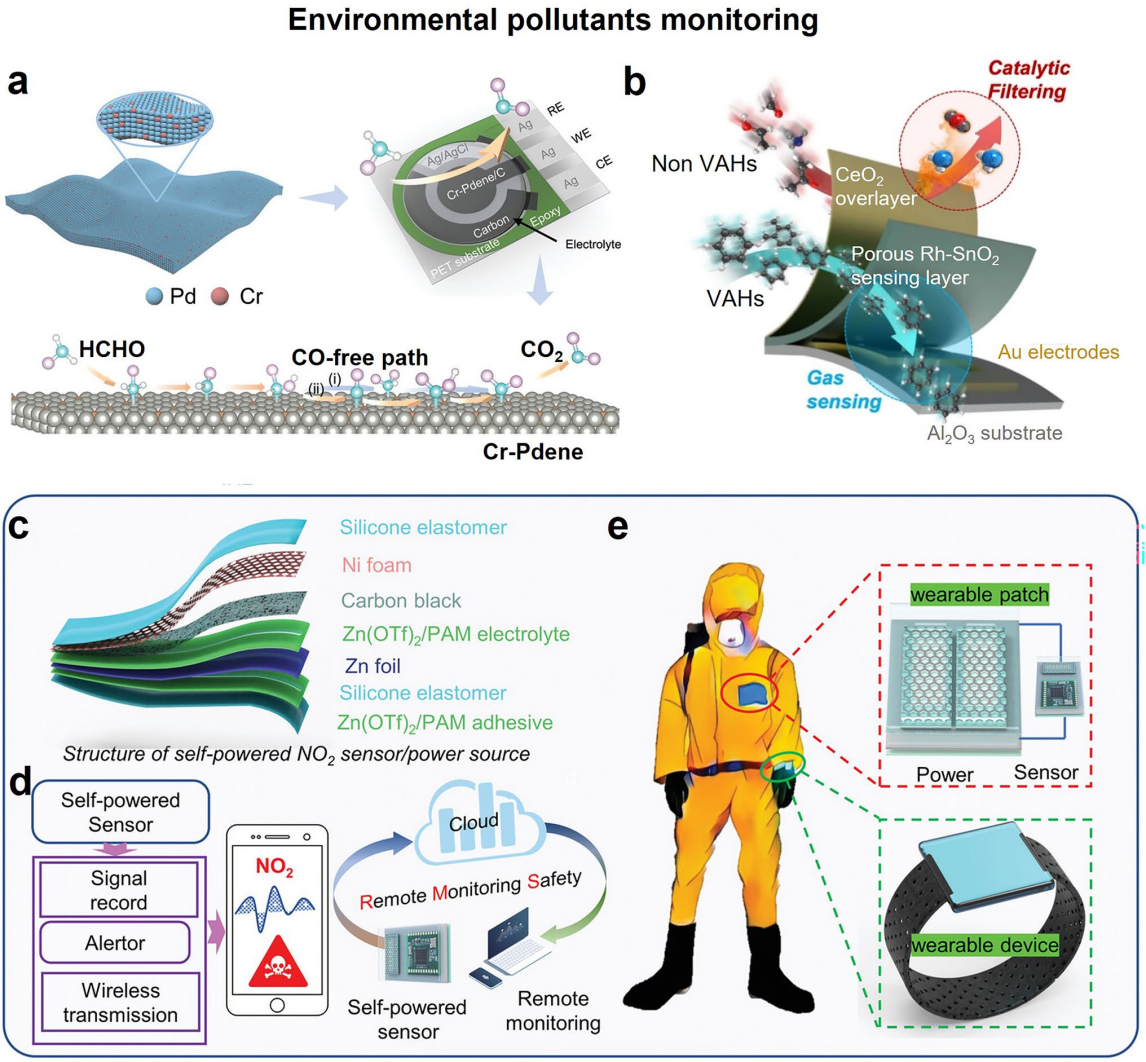
Flexible and wearable metal/metal oxide and hydrogel-based electronical gas sensors for environmental pollutants monitoring. **a** Formaldehyde monitoring with ultrathin Cr-Pdene layer. Reproduced with permission from Ref. [123]. Copyright 2022, Wiley–VCH. **b** Ultrasensitive discriminate VAHs and non-VAHs based on CeO_2_/Rh–SnO_2_ bilayer sensor. Reproduced with permission from Ref. [124]. Copyright 2023, Springer Nature. **c-e** Wireless self-powered NO_2_ gas sensor based on hydrogel patch. Reproduced with permission from Ref. [16]. Copyright 2023, Wiley–VCH

Formaldehyde (HCHO) is one of the most concerned indoor pollutants. It is considered a carcinogen upon long-term exposure to an environmental concentration exceeding 0.08 ppm, according to the World Health Organization [120]. Portable electronic gas sensors based on metal or metal oxide catalysts are usually used for selective detecting formaldehyde, owing to the formaldehyde oxidation reaction (FOR) [121, 122]. However, most of formaldehyde sensors cannot satisfy the international standard, and their long-term working stability can deteriorate via CO poisoning, a by-product of FOR. Guo et al. proposed a Cr-doped Pd-based electrochemical formaldehyde sensor to address these challenges (Fig. 5a) [123]. The sensing catalyst could selectively detect formaldehyde down to 72 ppb within 200 s via a highly efficient electrooxidation.

Volatile aromatic hydrocarbons (VAHs) are highly toxic trace air pollutants, including benzene, toluene, ethylbenzene, xylene, styrene, etc*.* Metal-oxide semiconductor-based chemiresistive sensors with high-operating temperature are favored in VAHs detection owing to the ability of adequate thermal activation to facilitate the sensing reaction between VAHs and surface oxygen species to induce charge transfer [124]. However, insufficient sensing selectivity and high-active interferent gas (such as ethanol and formaldehyde) restricted their practical application. Lee et al. recently reported a CeO_2_/Rh–SnO_2_ bilayer chemiresistive sensor array assisted with pattern recognition for distinguishing aromatic and nonaromatic gases (Fig. 5b) [124]. They revealed that the high VAHs sensing ability was ascribed to the CeO_2_ overlayer with moderate catalytic activity, which could covert highly reactive interfering gases to less- or non-reactive forms via catalytic oxidation.

Nitrogen oxide (NO_2_) is a common inorganic small molecule pollutant that causes acid rain and photochemical smog. Previous studies have reported diverse innovative electronic sensing materials for detecting trace NO_2_, in both rigid and flexible forms, such as TMDs [55, 125], MXenes [126], phosphorene [60], and MOFs [62]. One of the biggest challenges for NO_2_ chemiresistive and FET sensor is that the recovery difficulties owing to high physisorption energy and desorption problem and material deterioration in humidity. Another technique challenge for flexible NO_2_ sensors is their poor ductility under mechanical deformation in conjugation with flexible substrate. Wu et al. fabricated a flexible Zinc trifluoromethanesulfonate (Zn(OTf)_2_)/polyacrylamide (PAM)-carbon based NO_2_ electrochemical sensor to address mentioned challenges (Fig. 5c–e) [16]. The hydrogel-based sensor not only exhibited ultrahigh sensitivity (1.92%/ppb), ultralow limit of detection (LOD) of 0.1 ppb, outstanding recovery, but also worked well under different deformations and in subzero temperatures and under high humidity. They also integrated the hydrogel-based sensor into well-designed miniaturized circuit module to form a flexible wireless NO_2_ monitoring system, which could be worn for NO_2_ pre-warning.

### Medical and Healthcare Applications

**Fig. 6:**
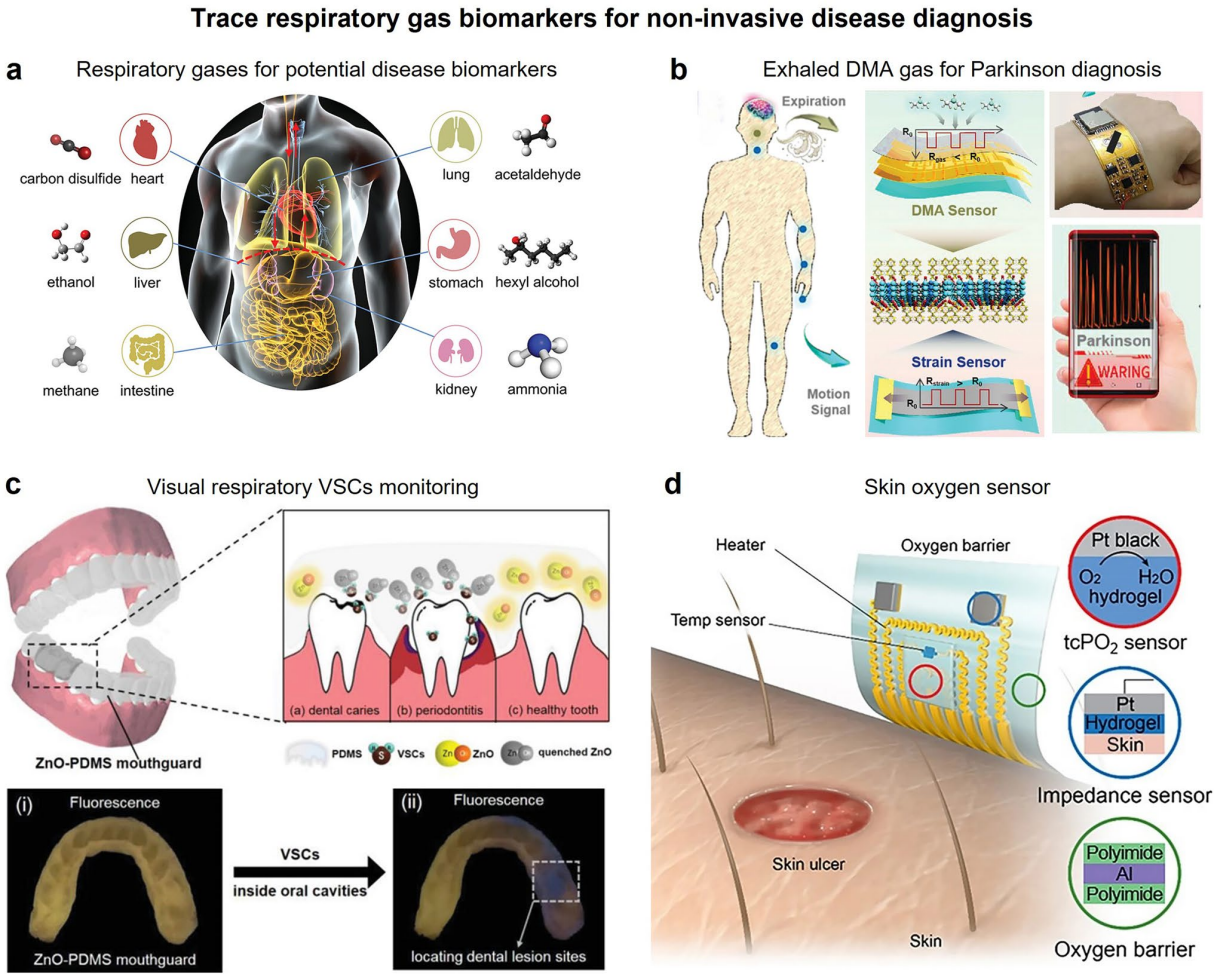
Multi-functional gas sensor of pathological biomarkers for disease diagnoses. **a** Representative exhaled biomarkers for pathological analysis. Reproduced with permission from Ref. [128]. Copyright 2021, Wiley–VCH. **b** Flexible dual-mode gas and strain sensor for point-of-care health-monitoring of Parkinson’s disease. Reproduced with permission from Ref. [132]. Copyright 2023, Wiley–VCH. **c** Wearable fluorescent mouthguard VSCs sensor for accurate localization of hidden dental lesion sites. Reproduced with permission from Ref. [134]. Copyright 2020, Wiley–VCH. **d** Flexible electronic skin oxygen molecules sensors. Reproduced with permission from Ref. [137]. Copyright 2021, American Association for the Advancement of Science

Point-of-care testing (POCT) is a on-site rapid sampling and instant assay method using portable analytical instruments and accompanying reagents that helps to shorten the clinical decision-making time [127]. Previous studies have confirmed that a variety of breathing [128], urine [129], and blood [130] volatiles could be utilized as the biomarker for early illness diagnosis (Fig. 6a). Thus, gas sensor-based POCT platform has a great potential as a rapid, inexpensive, noninvasive and painless method for early disease diagnosis and healthcare assessment [128, 130, 131]. However, weak changes in the biological signal generated in the early stage of diseases cannot be easily perceived, and the screening for single biomarker is not reliable for disease diagnosis. Thus, multi-functional sensors are on the demand for multidimensional and simultaneous biological signal acquisition. For instance, Zhou et al. fabricated a wearable healthcare platform using gas and strain sensing in non-overlapping mode for monitoring of abnormal physiological signals of Parkinson patients; the biomimetic sensing layer (ZIF-L@Ti_3_CNT_*x*_ composite: the zeolitic imidazolate framework flower-like particles in situ grown on the Ti_3_CNT_*x*_ nanosheet) was inspired by the synaptic structure (Fig. 6b) [132]. The bioinspired ZIF-L@Ti_3_CNT_*x*_-based sensor exhibited high performance in dual-mode monitoring of expiratory dimethylamine (DMA) gas markers and somatic kinematic dysfunctional tremors of Parkinson’s sufferers. With integration into a flexible circuit, the smart dual-mode sensor provides a prospect for real-time telemedicine Parkinson disease diagnosis. Zhang et al. also provided a POCT platform based on electronical array and machine learning for noninvasive disease diagnosis via urinary volatile, which will be discussed in a later section [129].

Dental caries and periodontitis are usually ascribed to food impaction and residues that easily breeding anaerobic bacteria to destruct periodontal tissue, accompanied with NH_3_ and volatile sulfur compounds (VSCs) emission from proteins metabolisms of these anaerobic bacteria [133, 134]. Exhaled volatile biomarkers have been popularized in oral disease diagnosis based on electrochemical sensing signal along with optical analysis. For example, Li and coworkers used a fluorescent material to visually identify the precise location of lesion sites by selectively detecting the emission concentration of local VSCs (Fig. 6c) [134]. Kim and coworkers developed a visual wearable sensor to detect the trace breath H_2_S of halitosis’ sufferers [135]. Jin and coworkers fabricated a local NH_3_ sensor array for halitosis diagnosis [136]. Multiple detecting methods provide preliminary diagnosis of dental disease.

Another emerging wearable bioelectronic devices is tissue-like skin-sensor. Kim et al. recently proposed a wearable bioelectronic skin-device formed by an ultrathin conductive functionalized hydrogel, which enables the rapid diffusion and transport of target bioanalytes (Fig. 6d) [137]. The hydrogel not only allowed the penetration of oxygen molecules from the blood vessels through skin, but also measured the reduction bioelectronic signals of diffused oxygen, providing a new way for transcutaneous oxygen pressure (tcPO_2_) measurement.

### Agricultural Quality Control

**Fig. 7:**
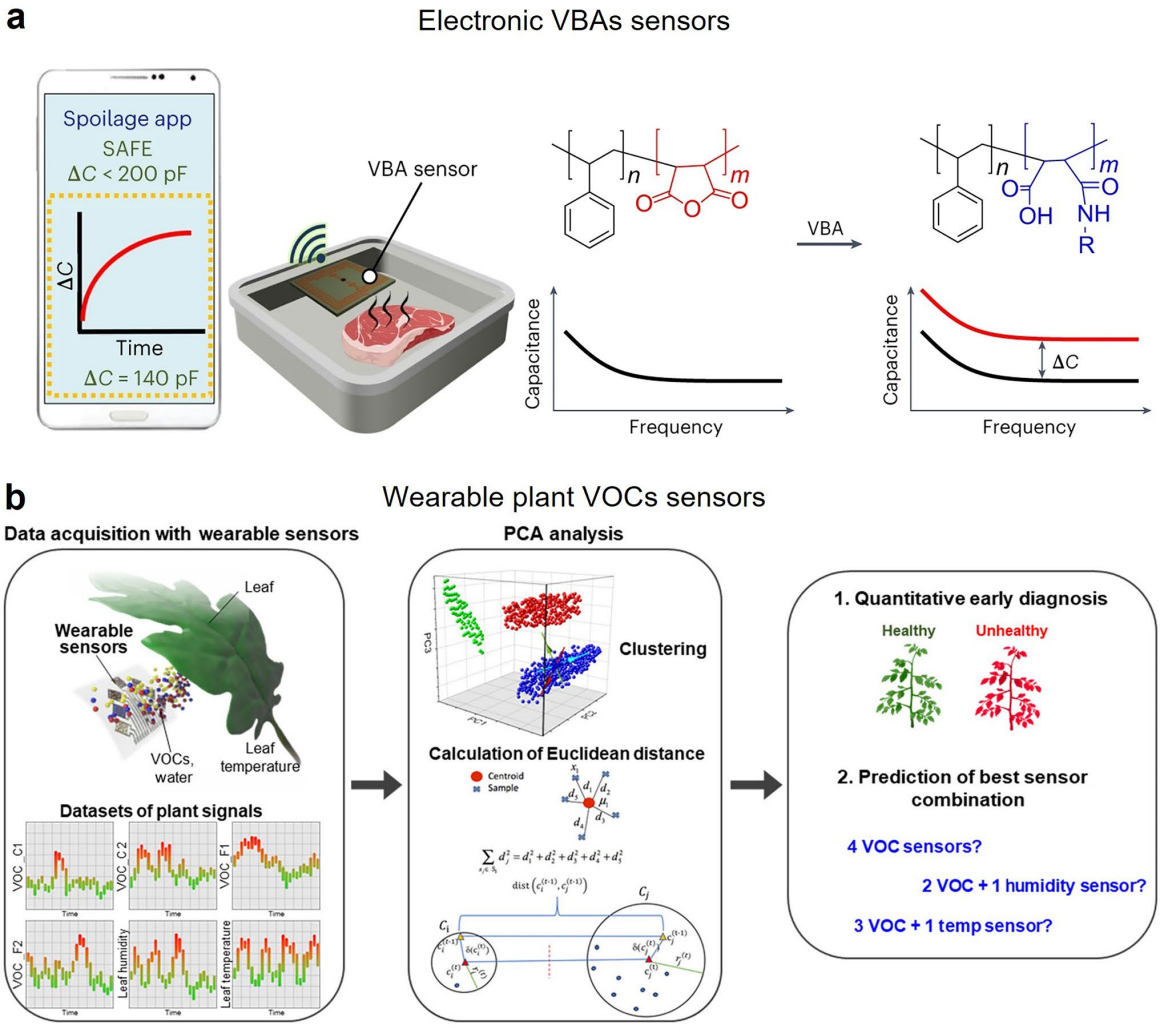
Smart gas sensors for food freshness and spoilage of protein-rich food. **a** Schematic illustration of real-time wireless monitoring of the biogenic amines released from spoiled meat. Reproduced with permission from Ref. [148]. Copyright 2023, Springer Nature. **b** Scheme illustration of wearable plant sensing platform integrated with VOCs, humidity and temperature sensors. Reproduced with permission from Ref. [152]. Copyright 2023, American Association for the Advancement of Science

Smart agricultural quality assessment sensors have attracted great interests as sensing platforms for real-time monitoring food freshness and spoilage for in situ storage and ex situ supply chains. Food spoilage sensors can report a spoiler alarm by detecting meat decomposition biomarkers such as total volatile basic nitrogen (TVBN) including volatile biogenic amines (VBA) and ammonia, produced by the decarboxylation of amino acids under interaction with microbes within protein-rich food [138–140]. Common VBAs include putrescine, cadaverine and spermidine, *n*-hexyl amine, benzylamine, NHEt_2_ [141–143], etc*.* Although previous study has reported lots of VBA sensors utilizing colorimetric and fluorescent changing method [142–147], their practical usage was limited in trace concentration detection with high-resolution optical camera. To address this challenge, Istif et al. invented a miniature (2 × 2 cm^2^) capacitor sensor based on poly(styrene-co-maleic anhydride) (PSMA) polymer sensing material for VBA response (Fig. 7a) [148]. Three aspects supported the advances and practicality of this type of VBA sensors: first, the low-cost and batch-fabrication-compatible of facile synthesis of PSMA; second, the miniaturization of the capacitor sensor for easy integration; and third, the compatibility of capacitive sensor with wireless mobile phones that not be impacted by motion artifacts.

Wearable plant sensors are one of ten emerging technologies in the world for improvement world food security [149–151]. Wei et al. provided an unprecedented multifunctional and real-time wearable plant sensor that could simultaneously measure plant VOCs, temperature and humidity (both leaf surface and the surrounding environment). They also first employed machine learning (such as PCA method for reduced data dimensions for classification) to process sensing data for quantitative early diagnosis and prediction of the best sensor combination (Fig. 7b).

### Public Safety

In recent years, there has been an increasing ecological and public health concern associated with domestic gas leakage, dangerous chemicals transportation wrecks, industrial accidents, natural disaster and national security. Particularly, major chemical leakage accidents, such as the derailed train carrying vinyl chloride hazardous chemicals in Ohio, frequent chemical plant explosions, and volcano eruptions, not only cause potential explosions and fire incidents by combustible substances, but also long-term health risks by leaked chemicals. Besides, chemical warfare agents (CWAs) used in military operations can cause huge damages to the human body through pathophysiological effects [153]. Therefore, great commercial demands have emerged for rapid analysis of toxic gas leak, flammable and explosive gases as well as nerve agents.

#### Gaseous Explosive Gas Sensors

**Fig. 8:**
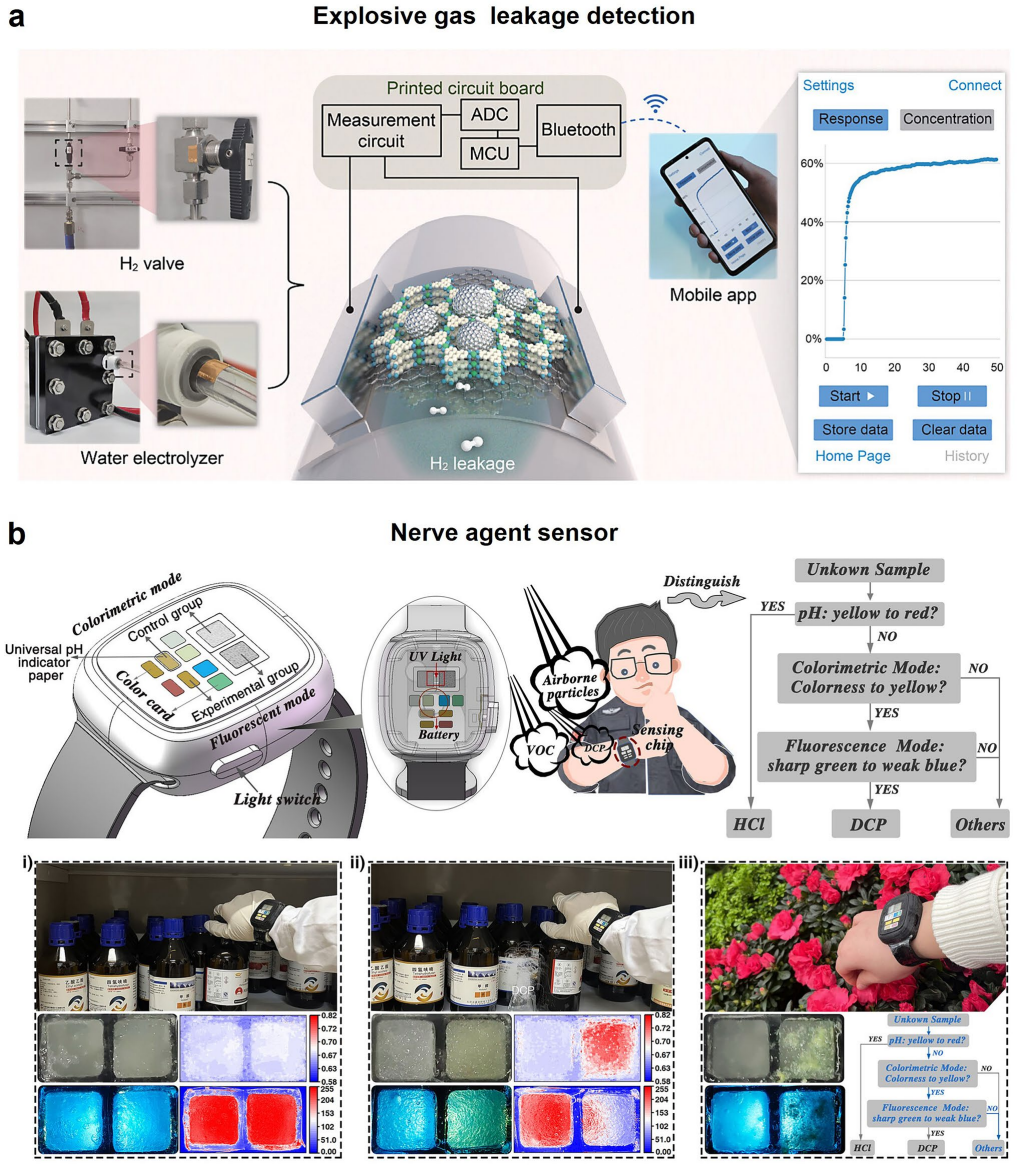
Smart explosive and nerve agent sensors for public safety. **a** Schematic diagram of a palladium-modified epitaxial metal–organic framework hydrogen sensor. Reproduced with permission from Ref. [158]. Copyright 2024, American Chemical Society. **b** Schematic diagram and logical discriminant diagram of a portable sensing platform for dual-mode recognition of the vapor of nerve agent analog DCP vapor. Reproduced with permission from Ref. [163]. Copyright 2024, American Chemical Society

Hydrogen (H_2_) has emerged as one of the most promising clean substitutes for fossil fuels. Hydrogen sensors are in high demand for safety management during transportation and utilization of H_2_ owing to its low ignition energy and wide explosive limits (4%-75%) [154]. Palladium is usually considered as the best noble metal (*e.g.*, Pd-Au dual-metal-modified In_2_O_3_ [154], Pd nanotube arrays [155], Pd NPs decorated graphene [156]) for H_2_ sensors because it converted into PdHx according to the reversible reaction of 2Pd + *x*H_2_ ↔ 2PdH_*x*_ [157]. To address the long-response time problem resulted from the flexible metal oxide semiconductor (MOS)-based sensors, Sun et al. recently designed an Pd-modified MOF thin film (MOF-Pd) and integrated it into a paper-based circuit for a fast H_2_ leakage detection (Fig. 8a) [158]. The Epi-MOF-Pd sensor is both flexible and enduring, demonstrating high sensitivity toward 1% H_2_ with 155% resistance response within 12 s over 10 thousand bending cycles.

Nitroaromatic explosives including picric acid (PA) and o-Nitrophenol (o-PN) are not only with great damage to people lives and property, but their residues contaminate natural resources and lead to human health and environmental sustainability. Optoelectronic sensors are popular in detection explosives including nitro-organics, nitramines, and peroxides, owing to their flexible and versatile chemical selectivity toward various explosives as a result of multiple analyte binding mechanisms [159]. Yang et al. also proposed an effective portable ultrasensitive dual-mode fluorescent sensor based on 3,4-bis (4-(1,2,2-triphenylvinyl) phenyl) thiophene (TPE-Z) hydrogel and a methyl red design concept for on-site detection of PA vapor [160], providing an innovative potential for on-site optoelectronic gas sensors.

#### Nerve-Agent Vapor Detection

CWAs including were intentionally developed for military targets and globally banned through the chemical weapons convention. Unfortunately, the low fabrication costs and easy manufacture, CWAs have still been used in some terrorist-related conflicts and resulted in mass civilian casualties. The organophosphorus nerve agents, such as diethylchlorophosphate (DCP), Sarin, Tabun and Soman, are extremely toxic to the human through the respiratory tract and skin [161]. The intake of trace nerve agents can result in an accumulation of acetylcholine in the central and peripheral nervous system, causing the destruction of the nerve impulses conduction and hence death within a few minutes. The biggest challenge for detection of nerve agents is that they are usually colorless and odorless, and one of the most popular effective ways for rapid onsite identification is by visual optoelectronic sensors, due to the low cost, ease fabrication and intrinsic optical properties. Huh et al. lately summarized a series of utilizable compounds including polymers, enzymes, organic or inorganic dyes and nanoparticles as colorimetric and fluorescent sensing materials [162]. Dou et al. recently proposed an innovative aggregation-induced emission (AIE) probe regulation strategy for an aggregated-to-aggregated colorimetric-fluorescent dual-mode for DCP vapor detection (Fig. 8b) [163]. They constructed a porous polymer-based chip loaded with the probe toward DCP vapor, integrating it into a watch, and achieved two-week continuous monitoring of DCP with an immediate response and low LOD down to 1.7 ppb. Atmosphere interferences such as aromatic compounds, esters, amines, alcohol, and carboxylic acids can passivate the sensors or cause to false-positive responses to nerve agents [164]. Shaw et al. [165] recently reported a fluorescence-based method for rapid differentiation of V-series and G-series nerve agents and successfully avoided false positive signal resulting by common acids.

## Smart Gas Sensors with Artificial Intelligence and Wireless Telecommunication Technology

**Fig. 9:**
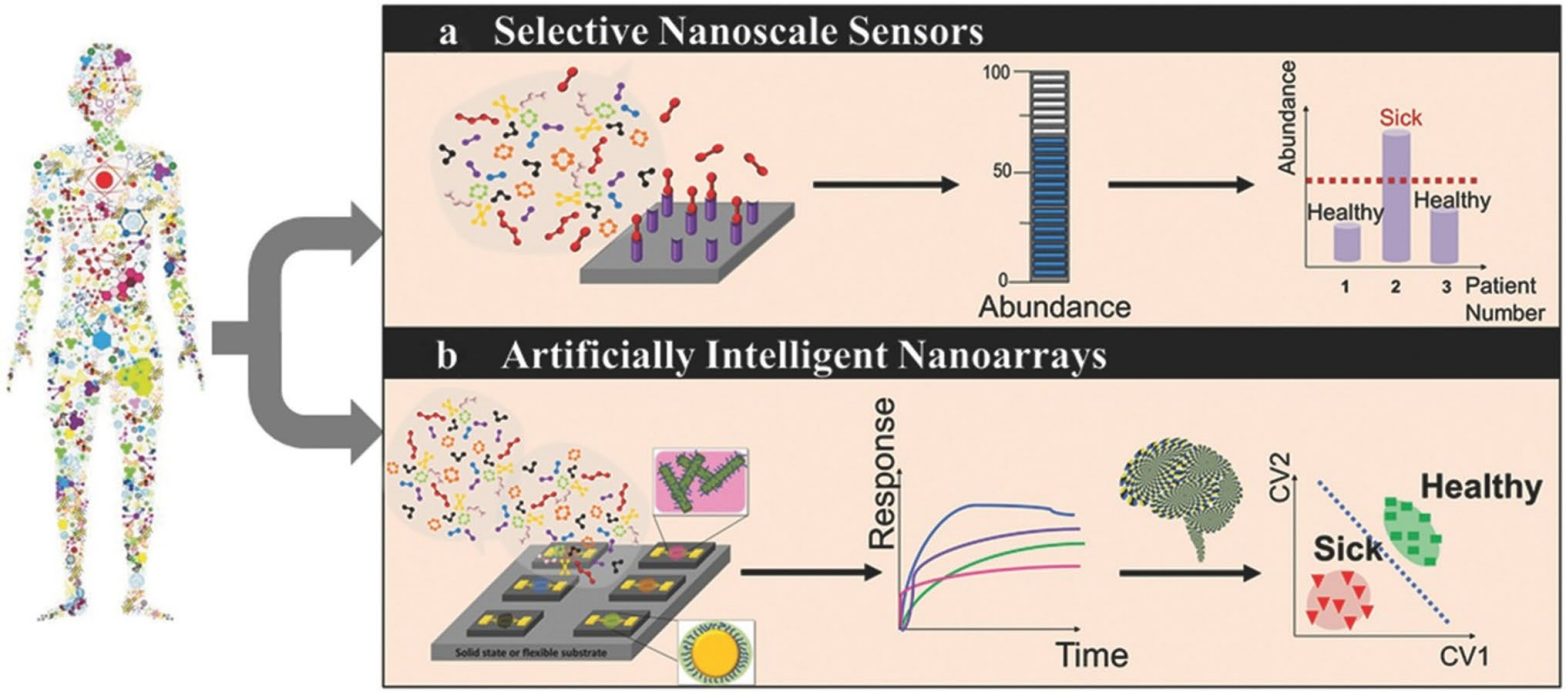
Scheme illustration of two target sensing modes (take the disease detection as examples): **a** selective nanoscale sensing mode; and** b** cross-reactive sensing mode. Reproduced with permission from Ref. [166]. Copyright 2015, Wiley–VCH

From the aforementioned newest state of gas sensors, the advanced materials, fabrication and integration techniques and data analysis are in high demand for design of selective gas sensors for practical uses. In fact, most present gas sensors, especially electronic gas sensors, simply focus on improving the selectivity toward target, with great challenges of cross-reactive sensitivity to address. Haick et al. have summarized the selective identification approaches: selective sensors and cross-reactive (or semi-sensitive) sensors combined with pattern recognition (Fig. 9) [166].

### Selective Sensor

Selective sensing mode focused on the high identification of specific target analyte in the presence of interference species, which usually requires the design of highly selective receptor materials to probe signals. Selective sensing generally defined as detecting specific gas in the presence of interfering gaseous species, ascribed to a more specifical analyte–sensor interaction. Till now, most reported selective sensors have focused on quite reactive small inorganic molecules such as NO_2_ [62], H_2_ [167], H_2_S [168], NH_3_ [169] and some VOCs (*e.g.*, formaldehyde [170, 171], acetone [172]). However, selective sensing is challenging for less reactive target analyte and even more challenging in complex mixtures, viz. effective discrimination among gases with chemical, structural, and electrical similarities [107].

### Sensor Array with Artificial Intelligence Algorithms

**Fig. 10:**
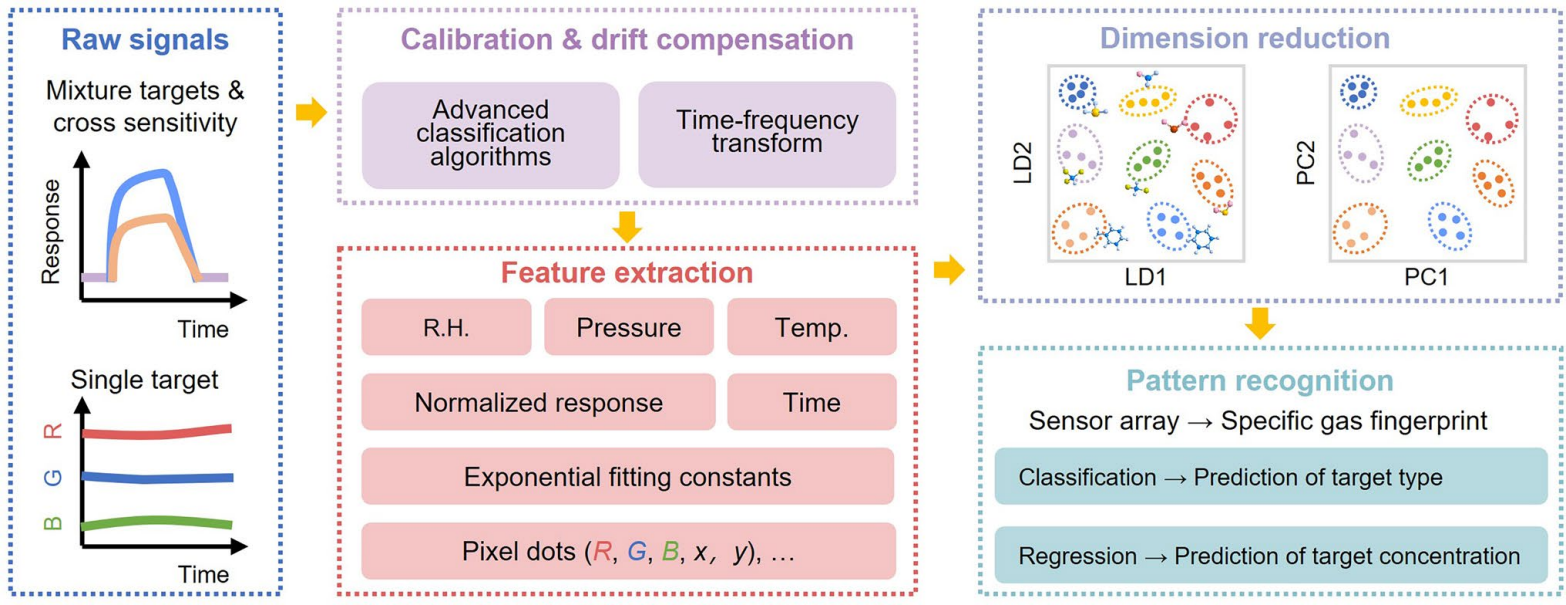
Overall artificial intelligent process from raw signals, calibration & drift compensation, feature extraction, dimension reduction to pattern recognition. R.H. for relative humidity and Temp. for temperature

Cross-reactive detection usually originates higher-reactive interferences that reduces the signals between target and sensing material (Fig. 10). It is preferable and used in a changing and unknown complex mixture analysis since most analyte–sensor interactions are based on less specific physical absorption [107]. The prevalent strategy for precise gas pattern recognition is by well-designed sensor array with assisted advanced artificial intelligence algorithms. The optimum sensor array should comprise of diverse high-specificity sensors and sensors that have individual responses (not strictly selective) to nearly all species in the targeted mixture [107]. All the responses are collected to produce analyte-specific response fingerprints and are analyzed by machine learning algorithms [173].

Additionally, the changing environment (*e.g.,* relative humidity, operating temperature, pressure) poses a threat to aging effect of sensing material, followed by response degradation (known as the drift error), which impacts the long-term stability. ML indeed paves an effective strategy for selective distinguishment of desirable gases by analysis of interfering effects and background noise in data analysis, and improving the long-term drift compensation of the sensor array via a transfer learning approach [174].

The core problems of machine learning can be divided into two broad categories, classification and regression. Before training, features engineering is the most prerequisites for model training and prediction accuracy. Feature engineering is the most important prerequisites for model training and prediction accuracy. For electronic sensors, normalized response signals, concentration, response/recovery time, area under the sensing curves, carrier mobility, threshold voltage, gate voltage, exponential fitting constants, and relative humidity are popularly extracted to be the classic features of a specific target species. For optoelectronic sensor, the R, G, B values as well as the coordinates within the pixel dots, which denoted as (*R, G, B, x, y*), and corresponding relative variations of the fluorescent intensity, chain length of targets are usually extracted as the features of targets. Model training requires a large dataset, sometimes principal component analysis (PCA) for feature selection, and accurate prediction of target type and concentration by choosing proper classification and regression algorithms.

Classifiers are used to predict gas types. For example, unsupervised learning algorithms (*e.g.,* principal component analysis [175], hierarchical clustering analysis [176], and K-means clustering [177]) are favorable in pre-classification of unknown species without labels, and supervised learning algorithms (*e.g.,* decision tree [178], random forests [179], support vector machine [179], K-nearest neighbors [180], and linear discriminant analysis [181]) can be used in accurate recognition of target species within the mixture, especially discrimination of VOCs with similar chemical structures. PCA is the most used approach to reduce dimensions and forms unlabeled clustering of targets, while linear discriminant analysis (LDA) is rarely used but can provide labeled classification of the targets [182].

Regressors are used to predict the gas concentration. For example, backpropagation neural network and extreme learning machine exhibit good performance in gas concentration estimation [183]. Linear regression is often used to solve problems when the dependent variable is a linear combination of the independent variables. While facing some more complex relationship, neural networks (*e.g.*, convolutional neural network [183], multilayer perception [179], recurrent neural network [184], radial basis functional neural network [185], and spiking neural network [186]) have been developed either through supervised learning or unsupervised learning. For example, artificial neural network (ANN) model is useful for estimation of the concentration of VOC mixtures in the same head group (*e.g.*, alkane chain length and molecular chain with hydroxyl/carboxyl/phenyl group) [187]. It also provides a major advantage in computational prediction speed in signal recovery and can be readily integrated into mobile terminals, paving the way for cost-effective and powerful sensing systems [188].

**Fig. 11:**
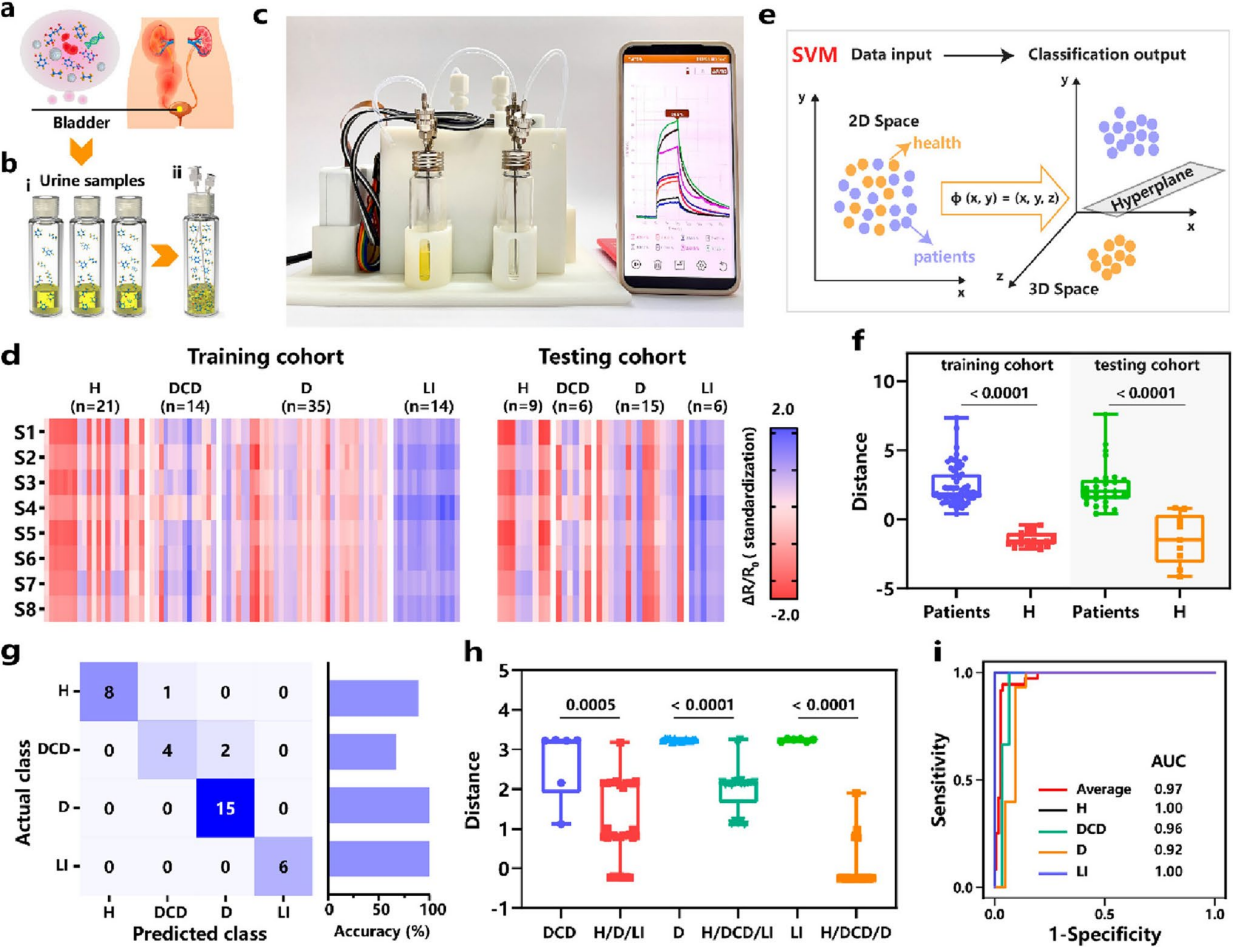
A portable POCT platform for noninvasive disease diagnosis via urine VOCs with ML. **a–c** Schematic illustration of urine sample collection and the testing process of MXene-based POCT platform. **d** Heatmap of electrical signals of the sensor array for the training cohort. **e** SVM algorithm helps to classify the health and the patients. **f-i** Effect verification of the prediction results from the POCT platform to identify the health and the patients with different diseases. Reproduced with permission from Ref. [129]. Copyright 2022, American Chemical Society

Deployment of machine learning (ML) algorithms enables the high prediction accuracy of unknown species recognition (Table 2). Moving on to the unfinished example in Sect. 3.2 related to urinary volatiles based POCT platform, Zhang et al. constructed a portable POCT platform integrated MXene frameworks-based cross-reactive sensor array (Fig. 11) [129]. They prepared MXene frameworks (MF) sensing layers via metal ion-doped, sequence-regulated and optimized ligand-engineered modifications, and produced differentiated responses patterns of 13 urinary volatiles via the MF sensor array. And they considered that the SVM algorithm owns the best diagnostic performance in discriminating the health and patient samples and a good classification of different disease (*e.g.*, diabetic comorbid depression, diabetes and liver impairment), among the other three ML algorithms. Their POCT platform achieves noninvasive diagnosis of several disease with a high accuracy of 91.7%.

**Table 2:** Applications of gas sensor/sensor array using various machine learning algorithms

Application	Gas type	Sensor number	ML method (processing task)		Refs
			Method	Processing	
Environmental monitoring	Indoor VOCs	5	PCA/ANN/CNN/DNN	Clustering, classification, prediction	[175]
	Indoor VOCs	1	RF/SVM/NB/MLP	Classification	[179]
	n-butanol	12	KNN/SVM	Classification	[180]
	VOCs	1	PCA/KNN/SVM/RF/LDA	Dimension reduction, classification	[181]
	Indoor air	1	GAT/KSS/RWLS/BPNN/MLP	Calibration, classification, prediction	[189]
	7 harmful gases	1	PCA/HCA/SVM	Dimension reduction, clustering, regression, prediction	[176]
	Contaminated air	8	PCA/LDA/RF/KNN	Dimension reduction, classification,	[190]
	VOCs	1	ANN	Classification, regression	[187]
	VOCs	1	KNN/SVM/SHBP	Classification	[191]
	VOCs	1	PCA	Dimension reduction	[192]
	Ethanol, acetone	8	PCA/LDA	Dimension reduction, classification, regression	[193]
	VOCs	6	SVM/KNN/MLP/RF/XGBoost/LGBM	Classification	[194]
	Toxic gases	10	LDA/SVM/MLP/DNN	Classification	[20]
	CO, NH_3_, NO_2_, CH_4_, and acetone	8	CNN	Classification, prediction	[195]
	Gaseous pollutants	15	HCA/PCA/SVM	Clustering, dimension reduction, classification, predication	[196]
	VOCs	2	PCA/KNN/pN-BPNN/SVM	Dimension reduction, feature extraction, classification, prediction	[197]
Disease diagnosis & health treatment	Lung cancer biomarkers	9	PCA	Dimension reduction, clustering	[198]
	Gastric cancer biomarkers		DFA	Classification	[199]
	Stable coronary artery disease biomarkers	19	SVM/KNN/ANN	Classification	[200]
	Ethanol	32	Self-developed algorithm/GPD	Classification, regression	[201]
	Breath biomarker	4	PCA	Classification	[202]
	Breath biomarkers	6	HCA/PCA/SVM/PLS	Clustering, classification, regression, prediction	[203]
	Allergic rhinitis biomarkers	32	PCA/CDA	Dimension reduction, clustering, classification	[204]
Food processing	Aflatoxin contamination (in maize)	12	SVM/KNN	Classification	[205]
	H_2_S (from eggs)	1	ANN	Classification	[206]
	Food sample odors	16	PCA/KNN/RF	Dimension reduction, classification	[207]
	Amine gases	2	PCA/HCA/CNN	Clustering, classification	[208]
Public safety	Chemical warfare agents	24	PCA/KNN/SVM/RF/LDA	Dimension reduction, classification,	[209]
	NH_3_, NO_2_	4	BP-NN/PLS/MLR	Classification, regression	[210]

*ANN* Artificial neural network; *AR* Allergic rhinitis; *BPNN* Back propagation neural network; *CDA* Canonical discriminant analysis; *CKD* Chronic kidney disease; *CNN* Convolutional neural networks; *DFA* Discriminant factor analysis; *DM* Diabetes mellitus; *DNN* Deep neural networks; *DT* Decision trees; *GAT* Global affine transformation; *GPD* Gaussian plume dispersion; *HCA* Hierarchical cluster analysis; *KNN* k-nearest neighbor; *KSS* Kennard–Stone sequential; *LDA* Linear discriminant analysis; *LGBM* Light gradient boosting machine; *LR* Logistic regression; *MLR* Mixed logistic regression; *MLP* Multilayer perceptron; *NB* Naïve Bayes; *NN* Neural network; *PCA* Principal component analysis; *PLS* Partial least squares regression; *QDA* Quadratic discriminant algorithm; *RF* Random forest; *RWLS* Robust weighted least square; *SCAD* stable coronary artery disease; *SHBP* single-hidden-layer back propagation artificial neural network; *SNN* Spiking neural network; *SVM* Support vector machine; *XGBoost*: Extreme gradient boosting

**Fig. 12:**
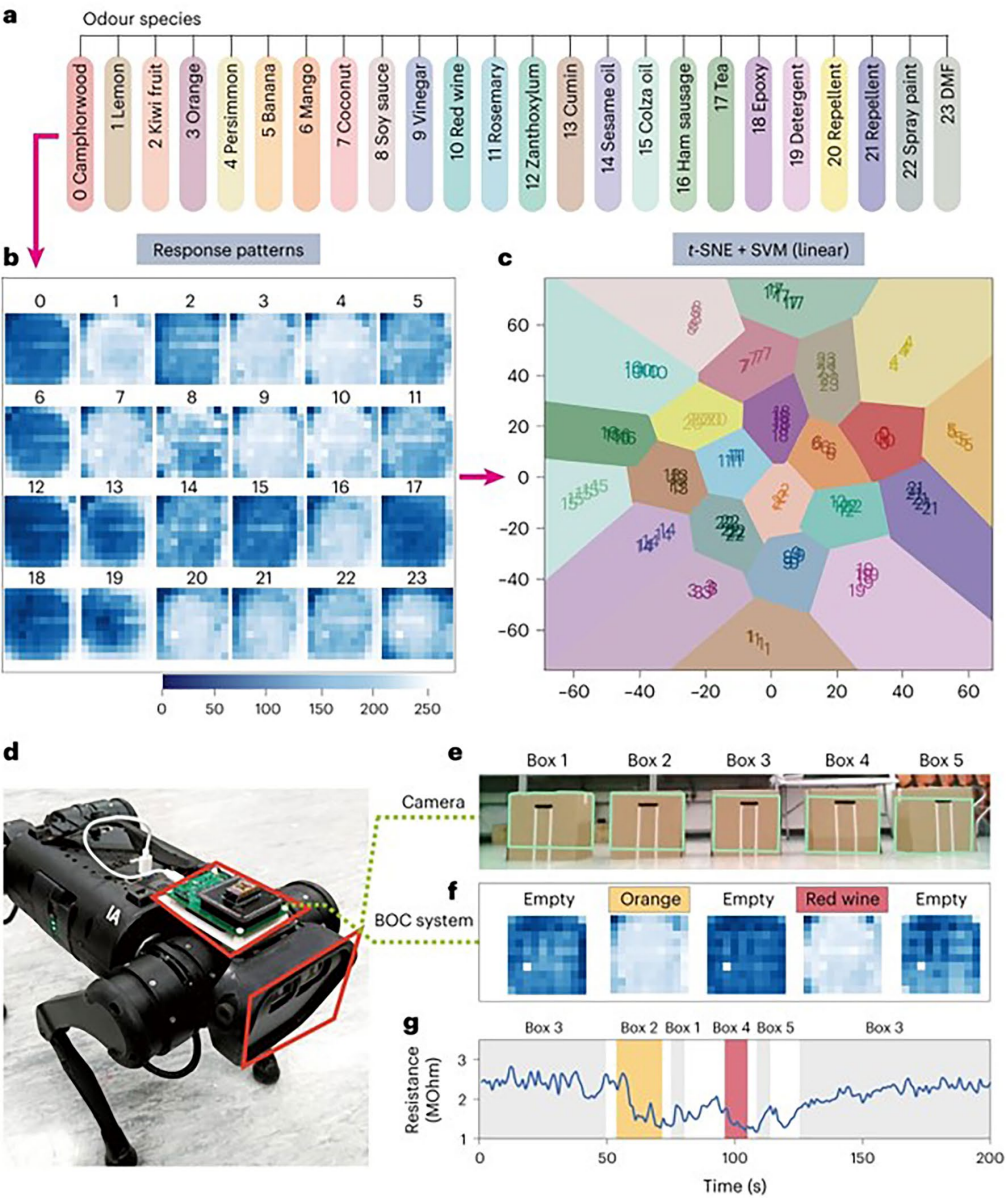
A Biomimetic olfactory system integrated into a quadrupedal mobile robot. The system uses a monolithic 3D PdO/SnO_2_ sensor-array chip, and per chip is cooperated with 10 thousand individually addressable sensors. **a–c** Cross-reactive sensitivity and artificial intelligence algorithms of the chips for distinguishability for 24 odors in mixed components. **d–g** The olfactory chips combined with vision sensors on a robot dog. Reproduced with permission from Ref. [212]. Copyright 2024, Springer Nature

The current trend for precise multi-gas identification and analyte distinguishment is using arrays of broadly cross-responsive sensors in conjugation with machine learning algorithms. Fan et al. recently reported a biomimetic olfactory chips (BOCs) system based on a high-density monolithic 3D PdO/SnO_2_ sensor array (100–10,000 sensors per chip), which mimicked the diversity of biological olfactory receptors (namely, the pixel diversity) [212]. It was supported by a peripheral signal read-out circuit and the resistance of each pixel could be read out accurately. Each pixel responded differently upon exposure to various odors; thus, the pixel diversity generated a series of signature patterns for various odor molecules. First, they examined that the BOC system could recognize 8 odors with different concentrations under a set of humidity background, and the prediction accuracy was up to 99.04% by using a CNN model for classification (predicting gas type) and regression (predicting gas concentration). Then, they collected 100 gas response patterns of each odor species of 24 odors (Fig. 12a, b) and recognize each odor in the gas mixtures with a t-distributed stochastic neighbor embedding and SVM algorithm (Fig. 12c). The excellent classification capability of the BOC system helps to effectively identifying orange and red wine from the blind boxes (Fig. 12d–g), demonstrating its immense potential of practical applications.

**Fig. 13:**
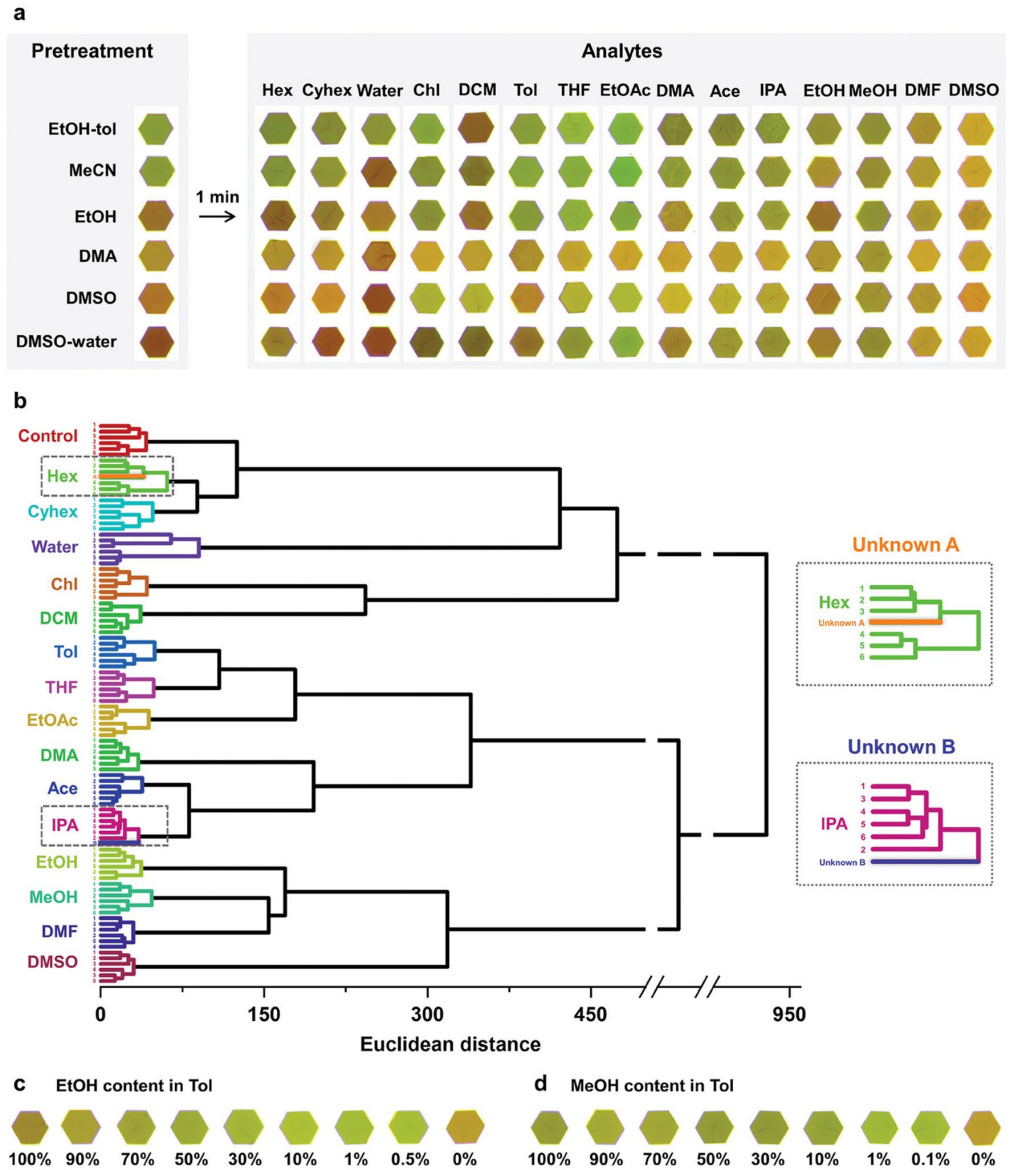
Colorimetric sensor array based on monolayer MOF films for VOC identification. **a** The color pattern before and after VOC analytes exposure. **b** Classification of similar analytes by HCA dendrogram. **c–d** Color variations of EtOH and MeOH in mixtures. Reproduced with permission from Ref. [211]. Copyright 2024, Springer Nature

One of the most prominent challenges for atmosphere gas detection is the humidity interference. One big advantage of optoelectronic sensor arrays is that they can be fabricated insensitive to humidity. Park et al. recently fabricated a novel colorimetric sensor array based on a series of 2D MOF films (DGIST-15) [211]. The monolayer film consisted of two moieties (dicopper paddlewheel clusters and dimethylamine azobenzene), showing a broad spectrum of colors from green to red that was sensitive to surrounding analyte species. The DGIST-15-based colorimetric sensor array was constructed by a series of DGIST-15 films pretreated by different solvents that responded to analytes with various color changes (Fig. 13a, b). The results showed that this sensor array exhibited diverse response patterns to 15 VOCs (including similar analytes such as Hex and Cyhex, IPA, EtOH, and MeOH, DMF and DMA). The array also exhibited the potential for identification of mixtures (Fig. 13c, d). Interestingly, they demonstrated that the DGIST-15 film exhibited real-time reversible color transitions in the varying RH of 10%–60%, thus required no extra heat treatment to displace the adsorbed water and facilitated continuous environmental monitoring.

### Smart Gas Sensors in “Internet of Things” Paradigm

IoT consists of smart devices that connect with each other by wireless communication, many different low power wireless communication technologies and protocols such as ZigBee, Bluetooth Low Energy (BLE), LoRa, SigFox, Z-Wave, WiFi, and Near Field Communication (NFC) can be used to connect the smart gas sensors for further data processing and future IoT applications. IoT-based early warning system for remote monitoring atmospheric air quality and epidemic events.

**Fig. 14:**
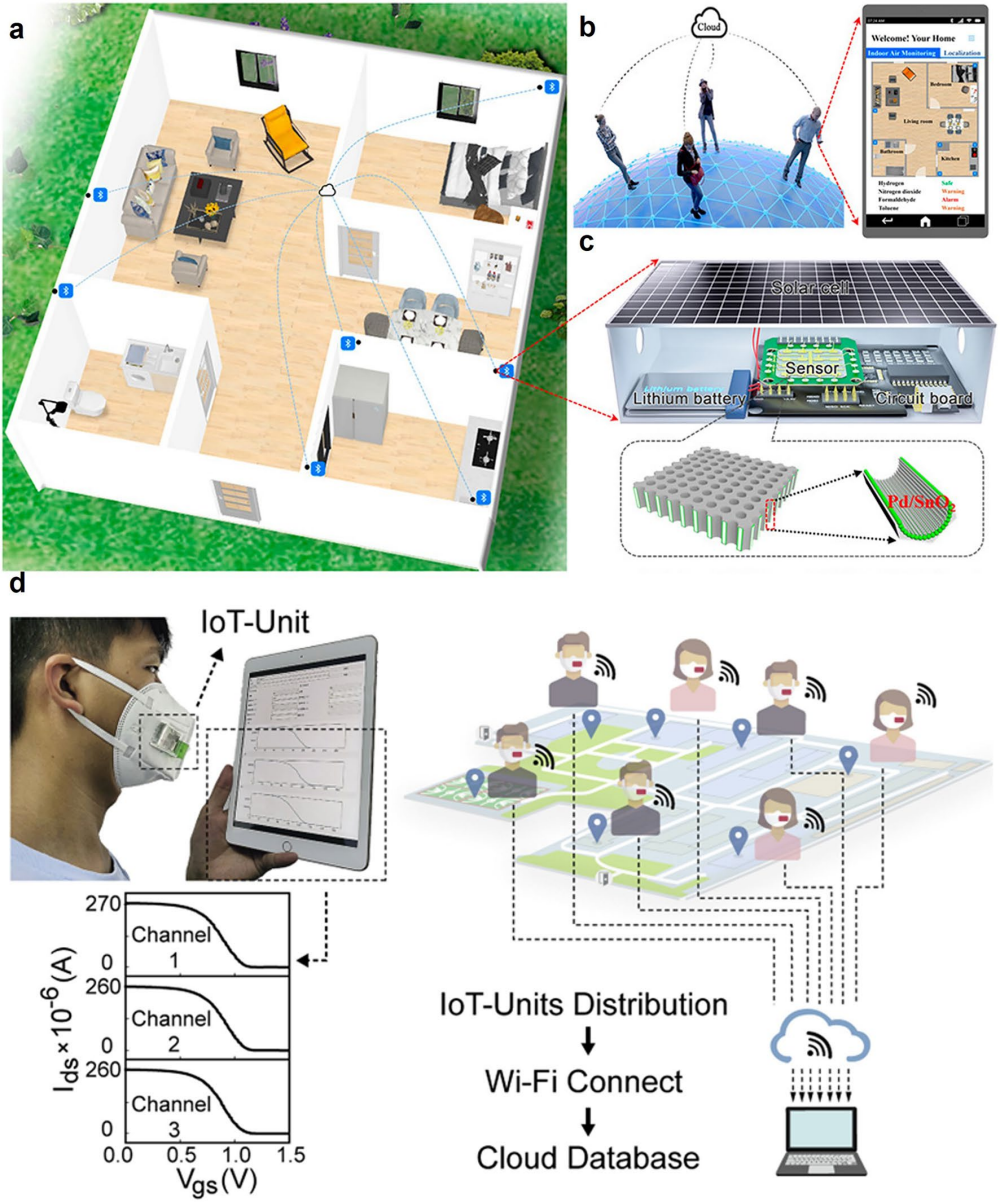
**a–c** Smart gas sensors in future smart home. Reproduced with permission from Ref. [9]. Copyright 2021, American Chemical Society. **d** Real-time monitoring platform of different respiratory infectious diseases for early warning of epidemic events. Reproduced with permission from Ref. [214]. Copyright 2022, Elsevier

Wireless sensing networks can improve the spatial and temporal resolution of the obtained sensing signals and support real-time detection in some inaccessible situations [85, 213]. For example, Fan et al. proposed a novel self-powered integrated nanostructured-gas-sensor (SINGOR) based on 3D Pd/SnO_2_ thin film and a wireless SINGOR network for building a smart home (Fig. 14a–c) [9]. The 3D Pd/SnO_2_ thin film-based sensor array with PCA and SVM algorithms was used for providing the accurate identification of H_2_, formaldehyde, toluene, and acetone in a wide range of relative humidity (0–85%) through cross-responses. Then, a series of SINGOR were deployed in the several sites of a house and generated a wireless sensor network for uploading their continuous monitoring data, and achieved accurate gas leakage localization. Jin et al. also designed a novel photoluminescence-enhanced light fidelity (Li-Fi) telecommunication technique for their NO_2_ sensors to achieve remotely tracking air pollutants change with high sensing performance and low-power consumption [18].

Wearable bioelectronic gas sensors in combination with IoT technology also present a future prospect for preventing outbreaks of respiratory infectious diseases. For instance, Wang et al. developed a wearable bioelectronic mask for wireless monitoring viral proteins from airborne media (Fig. 14d) [214]. With the assist of IoT technology, the smart masks were expected to warn and prevent epidemic events. Besides, AI-enabled database analytics at the cloud server realize new AIoT technology for low-cost collection and transformation of sensing information from the smart gas sensors to the cloud wirelessly. With visual data from the sensing device assisted by algorithms and statistical models tool, flexible H_2_ sensor still works well in extreme deformed condition [215].

## Summary and Prospects

In the past decades, smart gas sensor technology has been motivated by integration, IoT, and advanced algorithms and shifts from current rigid portable device to flexible and wearable electronics. The fundamental working principles of electrical and optoelectronic gas sensors, the full operation procedures of smart wearable gas sensors, the sensor architectures, and recent advances of smart gas sensors in a diverse range of applications are introduced in this review. With wireless telecommunication technique and machine learning-enabled technology to power IoT, gases can be well identified for diverse applications with high sensing performances. This section discusses the challenges and future prospects of smart gas sensors.

### Sensing Accuracy and Detection Discrimination

High level of accuracy and precision in electronic and optoelectronic measurement is the prerequisite for sensing reliability in practical monitoring. Sensor drift is the key factor that induces inaccurate electronic measurement, which is the result of the aging of the sensor component under long-term changing environment conditions (*e.g.*, temperature and humidity). Inaccurate colorimetric reading also impacts the interpretation of sensing results. To address these challenges, exploring and utilizing powerful calibration and analysis techniques, such as machine leaning and deep learning, is crucial for measurement error reduction and sensing dataset reproducibility. Although current smart gas sensors and sensor arrays show practical potential in multicomponents identification by pattern recognition methods, the complexity of air mixtures remains great challenge for accurate sesning. Deep-learning with big sensing data may provide an effective solution to address the sensing accuracy issue in a complex and diverse environment. 

### Data Integrity and Reproducibility

Conventional opinions of the reliability and credibility of gas sensors for practical usages are based on the data integrity and reproducibility; however, recent opinions proposed that adequate data processed with deep-learning have a great error rate, even with a better prediction of classification, which remains a question for exploration. Yet, with a small dataset, accurate, reliable and reproducible data across instruments and operators are still significant for the credibility of gas sensors in practical application.

### Low-Frequency Noise

One crucial parameter to impact the LOD of electronic gas sensors is the low-frequency noise (*e.g.*, 1/f noise and random telegraph noise) during sensing transmission to the computing unit, which has been overlooked in most studies. High SNR is a necessary parameter in the integrated system with massive integration of sensor array; otherwise, a higher low-frequency noise could severely degrade the sensitivity. However, it still remains a challenge to address this issue due to the lack of systematical investigation on the origin and generation mechanism of the sensor noise.

### Inherent Consistence

Sensor performances can endure inherent inconsistence across device or environment variations, when scaled up for larger fabrication. For example, the physisorption property of some 2D material (*e.g.*, black phosphorus has a wide thickness-tunable band gap) can be highly influenced by material configuration, which can induce device-to-device variations in the chemiresistors and FETs. Some promising colorimetric sensing materials such as anthocyanins vary from different biological sources and environment conditions. Though the signal normalization processing provides an effective way to suppress the device inconsistence, the essential innovate solutions are required to tackle these challenges, such as improving material stability and refining fabrication techniques.

### Material Science and Digital Integration

The trend of smart electronic and optoelectronic gas sensors is poised at the intersection of advanced material science and digital technology. The innovative sensing materials lay the foundation of sensitivity and selectivity. Spontaneously, the integration of IoT and AI-driven data processing technology revolutionize the collection, analysis, and utilization of sensing dataset. For electronic gas sensors, advanced neural network algorithms (*e.g.*, KNN, CNN) and time–frequency transform help calibrate and make drift compensation to improve long-term sensing performances. For optoelectronic gas sensors, advanced image processing algorithms (*e.g.*, thresholding and edge detection) are helpful for accurate colorimetry data extraction. Precise collection and analysis of the color change through rationally choose color models assist to accurately calculate analyte concentration and improve sensing reliability. AI technologies also improve data interpretation. For instance, SVM and MLP assist gas monitoring and early warning for environmental issues. Deep learning algorithms, especially neural networks, coordinated with optoelectronic sensors, enable food safety insurance by identifying complex patterns.

This review discusses the current development and future prospects of smart gas sensors. The latest interdisciplinary concept of smart gas sensors gathers the advancements of material science, embedded computing technology, wireless sensing network and IoT, which underscores the versatility and transformative potential.

## Supplementary Information

Below is the link to the electronic supplementary material.

Supplementary file1 (DOCX 28 KB)
