# Supplementary material for: Smart Gas Sensors: Recent Developments and Future Prospective
Source: Nanomicro Lett. 2024 Nov 4;17:54. doi: 10.1007/s40820-024-01543-w (PMC11532330; doi:10.1007/s40820-024-01543-w)
Supplement: Supplementary file 1 — Supplementary file1 (DOCX 28 KB) [file 40820_2024_1543_MOESM1_ESM.docx]

Supporting Information for

**Smart Gas Sensors: Recent Developments and Future Prospective**

Boyang Zong^1, 2^, Shufang Wu^3^, Yuehong Yang^1, 2^, Qiuju Li^1, 2,^ *, Tian Tao^1, 2^, Shun Mao^1, 2,^ *

^1^ College of Environmental Science and Engineering, Biomedical Multidisciplinary Innovation Research Institute, Shanghai East Hospital, State Key Laboratory of Pollution Control and Resource Reuse, Tongji University, 1239 Siping Road, Shanghai 200092, P. R. China

^2^ Shanghai Institute of Pollution Control and Ecological Security, Shanghai 200092, P. R. China

^3^ Microbiome Medicine Center, Department of Laboratory Medicine, Zhujiang Hospital, Southern Medical University, Guangzhou 510280, P. R. China

*Corresponding authors. E-mail: [qjli@tongji.edu.cn](mailto:qjli@tongji.edu.cn) (Qiuju Li); [shunmao@tongji.edu.cn](mailto:shunmao@tongji.edu.cn) (Shun Mao)

**Supplementary Table**

**Table S1** Key sensing parameters and definitions in gas sensors [S1-S6]

| **Parameter** | **Formula** | **Symbol definition** |
| --- | --- | --- |
| Sensitivity  (Chemiresistor) | $\text{Sensitivity=}\frac{{\text{Δ}\text{R}}_{\text{analyte}}}{\text{R}_{\text{0}}}\text{×100\%=}\frac{\text{R}_{\text{analyte}}\text{-}\text{R}_{\text{0}}}{\text{R}_{\text{0}}}\text{×100\%}$  $\text{Sensitivity=}\frac{{\text{Δ}\text{I}}_{\text{analyte}}}{I_{\text{0}}}\text{×100\%=}\frac{\text{I}_{\text{analyte}}\text{-}\text{I}_{\text{0}}}{\text{I}_{\text{0}}}\text{×100\%}$ | *R*_0_ and *I*_0_ are the initial resistance and current of the device under an applied voltage, respectively; and *R*_analyte_ and *I*_analyte_ are the resistance and current upon exposure to analyte, respectively. |
| Sensitivity  (Chemical diodes) | $\text{Sensitivity=}\frac{{\text{Δ}\text{V}}_{\text{analyte}}}{V_{\text{0}}}\text{×100\%=}\frac{\text{V}_{\text{analyte}}\text{-}\text{V}_{\text{0}}}{\text{V}_{\text{0}}}\text{×100\%}$ | *V*_analyte_ and *V*_0_ are the applied voltage at a constant current density with and without the analyte, respectively. If the devices are operated at constant voltage, the sensitivity can also be given by Δ*I*/*I.* |
| Sensitivity  (FETs) | $\text{Sensitivity=}\frac{\text{Δ}\text{I}_{\text{DS, analyte}}}{\text{I}_{\text{DS}}}\text{=}\frac{\text{I}_{\text{DS, analyte}}\text{-}\text{I}_{\text{DS}}}{\text{I}_{\text{DS}}}\text{×100\%}$ | *I_DS,_* _analyte_ is the current exposure to analyte and *I*_DS_ is the initial current at a constant gate-source and drain-source voltage. |
| Sensitivity  (Chemical capacitors) | $\text{Sensitivity=}\frac{\text{Δ}\text{C}}{\text{C}_{\text{0}}}\text{=}\frac{\text{C}_{\text{analyte}}\text{-}\text{C}_{\text{0}}}{\text{C}_{\text{0}}}\text{×100\%}$ | *C*_analyte_ is the capacitance exposure to analyte and *C*_0_ is the initial capacitance in farads at a fixed voltage. If the devices are operated at constant capacitance, the sensitivity can also be given by Δ*V*/*V*. |
| Sensitivity  (Colorimentric sensors) | $\text{Sensitivity=}\frac{\text{Δ}\text{C}}{\text{C}_{\text{0}}}\text{=}\frac{\text{C}_{\text{analyte}}\text{-}\text{C}_{\text{0}}}{\text{C}_{\text{0}}}\text{×100\%}$ | *C*_analyte_ is a calculated color signal, *C*_0_ is its initial state (without analyte). |
| Sensitivity  (Colorimetric sensors): Total color difference, or the CIE color parameter (Δ*E*) | $\text{Δ}\text{E}\text{=}\sqrt{{(L-L_{0})}^{2}+{(a-a_{0})}^{2}+{(b-b_{0})}^{2}}$ | *L*, *a*, and *b* are the initial color values of the sample; and *L*_0_, *a*_0_ and *b*_0_ are the color values of a standard whiteboard (*L*_0_=100, *a*_0_=0, *b*_0_=0). |
| Signal-noise ratio (*SNR*) | $\text{SNR}\text{min}\text{=}\text{S}_{\text{min}}\text{/}\text{N}_{\text{avg}}$ | *N*_avg_ is defined as the mean of the standard deviations of 300 data point in the baseline (without analyte); and *S*_min_ is defined as the minimum resolvable signal of the sensor. |

**Supplementary References**

1. J. Dai, O. Ogbeide, N. Macadam, Q. Sun, W. Yu et al., Printed gas sensors. Chem. Soc. Rev. **49**, 1756-1789 (2020). <https://doi.org/10.1039/C9CS00459A>
2. Z. Meng, R. Stolz, L. Mendecki, K. Mirica, Electrically-transduced chemical sensors based on two-dimensional nanomaterials. Chem. Rev. **119**(1), 478-598 (2019). <https://doi.org/10.1021/acs.chemrev.8b00311>
3. R. Paolesse, S. Nardis, D. Monti, M. Stefanelli, C. Di Natale, Porphyrinoids for chemical sensor applications. Chem. Rev. **117**(4), 2517-2583 (2017). <https://doi.org/10.1021/acs.chemrev.6b00361>
4. Q. Xu, B. Zong, Q. Li, X. Fang, S. Mao et al., H_2_S sensing under various humidity conditions with Ag nanoparticle functionalized Ti_3_C_2_T_x_ MXene field-effect transistors. J. Hazard. Mater. **424**, 127492 (2022). <https://doi.org/10.1016/j.jhazmat.2021.127492>
5. Z. Li, E. Tian, S. Wang, M. Ye, S. Li et al., Single-atom catalysts: Promotors of highly sensitive and selective sensors. Chem. Soci. Rev. **52**, 5088-5134 (2023). <https://doi.org/10.1039/D2CS00191H>
6. T. Minami, Y. Liu, A. Akdeniz, P. Koutnik, N. Esipenko et al., Intramolecular indicator displacement assay for anions: Supramolecular sensor for glyphosate. J. Am. Chem. Soc. **136**(32)**,** 11396-11401 (2014). <https://doi.org/10.1021/ja504535q>
